# Epidemiologic consequences of preclinical transmission of foot-and-mouth disease virus in cattle

**DOI:** 10.3389/fvets.2025.1651091

**Published:** 2025-08-29

**Authors:** Stormy Scharzenberger, John M. Humphreys, Columb Rigney, Alexis Freifeld, Carolina Stenfeldt, Jonathan Arzt

**Affiliations:** ^1^Foreign Animal Disease Research Unit, Agricultural Research Service, U.S. Department of Agriculture, Plum Island Animal Disease Center (PIADC) and National Bio and Agro-Defense Facility (NBAF), Manhattan, KS, United States; ^2^Transboundary Disease Analytics Unit, Animal and Plant Health Inspection Service, U.S. Department of Agriculture, Center for Epidemiology and Animal Health, Fort Collins, CO, United States; ^3^Department of Diagnostic Medicine/Pathobiology, Kansas State University, Manhattan, KS, United States

**Keywords:** foot-and-mouth disease, FMD, simulation modeling, cattle, preclinical transmission, transboundary diseases, epidemiology

## Abstract

Foot-and-mouth disease virus (FMDV) can be transmitted during the incubation phase, before clinical detection, but the epidemiological consequences of this preclinical infectious period have not been fully assessed in cattle. Using experimental data derived from transmission studies performed *in vivo*, we parameterized a state-transition model and simulated FMDV outbreaks across three U.S. regions under varying durations of preclinical infectiousness. We evaluated multiple epidemiologic outcomes under both optimal (1 day after clinical onset) and suboptimal (4 days after clinical onset) detection scenarios. The modeled output demonstrated that even a single day of preclinical transmission significantly increased outbreak magnitude, spatial extent, and duration. These effects were magnified under suboptimal detection and when simulating low-virulence virus strains with prolonged preclinical phases. Optimal response consistently reduced outbreak severity, with greater mitigation observed in the Eastern and Central U.S. as the preclinical phase lengthened. Our findings demonstrate that omission of preclinical transmission from FMD models results in systematic underestimation of outbreak impacts. Incorporating incubation phase transmission is essential for realistic epidemic forecasting, effective preparedness planning, and region-specific response prioritization.

## 1 Introduction

Foot-and-mouth disease (FMD) is a highly contagious viral disease (species: *Aphthovirus vesiculae*, family: *Picornaviridae*) that affects wild and domestic cloven-hoofed animals, including many species of agricultural importance (1–4). Susceptible livestock in the United States (U.S.) include cattle, pigs, sheep, goats, and farmed bison and cervids (5). The U.S. is also home to susceptible wildlife species, including mule deer, which were involved in the outbreak that occurred in California in 1924 (6, 7).

The classical clinical presentation of FMD is characterized by fever, lameness, and the development of vesicular lesions on the mouth, hooves, and teats (1, 8). These symptoms can be debilitating, resulting in reduced animal welfare and severe losses in productivity (1, 4). However, the clinical severity of FMD varies both depending on intrinsic factors associated with the specific virus isolate, and on the species, breed, and age of the affected host (1). Although mortality is generally low in adult animals, the morbidity rate is very high in naïve livestock populations (4, 9). Acute myocarditis can cause death sporadically in young animals and rarely in adults (10–13).

FMD remains one of the most substantial concerns for transboundary animal disease impact globally (2, 4). The foot-and-mouth disease virus (FMDV) is estimated to circulate in 77% of the world’s livestock population and remains endemic in several parts of Africa, most of the Middle East, and Asia (4). The World Organisation for Animal Health (WOAH) currently recognizes Australia, New Zealand, North America, Central America, and the majority of countries in South America as free of FMD (4). The Hemispheric Program for the Eradication of Foot-and-Mouth Disease (PHEFA) was established in 1988 and has continued to make progress toward the eradication of FMD in Latin America (14). However, FMD is a transboundary animal disease, and FMD-free nations remain under constant threat of an incursion (4). This is evidenced by the recent detections of FMD in Germany, Hungary, and Slovakia (15, 16). Furthermore, there are many countries in Europe and Asia that have maintained FMD-free status despite detection of FMD elsewhere in these continents (4).

Although FMD was eradicated from the U.S. nearly a century ago in 1929, FMD is still considered a high-risk disease. In the event of an FMD incursion into the U.S., anticipated economic consequences include production losses, potentially long-lasting restrictions on international trade, and expenses associated with the control and eradication of the disease (5). The economic impact of a simulated FMD outbreak originating on a large dairy farm in California was estimated at 2.3 to 69 billion USD when detection of the initial case was delayed (17). For these reasons, a rapid and coordinated response is necessary at the national level. Emergency response measures must be prompt and practical while accounting for important economic, sociopolitical, and animal welfare considerations.

FMDV exists as seven serotypes (O, A, C, Asia 1, SAT [Southern African Territories] −1, −2, and −3) with many distinct strains and lineages within serotypes that vary in antigenicity, host range, virulence, and infection dynamics (3, 18, 19). Consequently, outbreak characteristics vary greatly depending on the strain and the composition of the host species population affected (20). Despite the diversity of FMD viruses, the majority of FMD modeling studies to date are based on characteristics of a serotype O virus (20). Serotype O is the most prevalent and geographically distributed serotype; however, multiple serotypes often circulate concurrently (9, 20–22). Applying modeling parameters derived from a diversity of FMDV strains may improve our understanding of potential outcomes and greatly enhance preparedness by accounting for uncertainty and variability.

**Figure 1:**
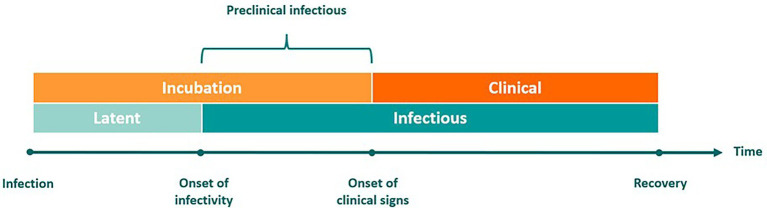
Defining distinct phases of infectious disease progression. Infection dynamics can be described relative to clinical signs of disease (represented by the orange bars on the top) or relative to the period of infectiousness (represented by the blue bars on the bottom). The incubation phase begins at the time of infection and ends upon expression of clinical signs. Infected individuals then transit into the clinical phase. The latent phase begins at the time of infection and ends at the onset of infectivity. When incubation is longer than latency, there is a preclinical infectious period that begins at the onset of infectivity and ends at the onset of clinical signs. The preclinical infectious period is the time during which incubation phase transmission may occur.

Disease transmission dynamics can be described relative to the onset of clinical signs or with respect to the period of infectiousness (Figure 1). The incubation period is the time between infection and the onset of observable clinical signs, during which the pathogen is replicating. By comparison, the latent phase refers to the interval between infection and the point at which the host becomes infectious and capable of transmitting the pathogen to others.

When transmission occurs before clinical signs appear, this is referred to as the preclinical infectious phase. This phase has a pivotal role in determining the severity of outbreaks. The terms preclinical and subclinical have been misused interchangeably; however, the concept of subclinical FMD is further complicated by the phenomenon of the FMDV carrier state in ruminants, which is another form of subclinical infection (23). The term subclinical refers to any animal infected with FMDV that does not currently exhibit clinical signs of FMD. This encompasses preclinical animals, FMDV carriers, and livestock effectively vaccinated against FMDV.

In the context of this work, we use the term preclinical to refer to cattle that are infected with FMDV, are still in the incubation phase, and have not progressed into the clinical phase. The assumption is that these animals will eventually develop observable signs of FMD. During the preclinical infectious phase, animals may transmit the virus in the absence of observable clinical signs and before control measures are implemented (24). Virus may also be detected via active surveillance by diagnostic testing of infectious animals prior to the observation of clinical disease. Once these animals progress into the clinical infectious phase, disease may be detected via passive surveillance (i.e., producer observation of clinical signs) and confirmed via diagnostic testing.

Despite a long history of research on FMDV, few published studies have examined the transmission of FMDV during the incubation phase (25, 26). One published work estimated that the preclinical infectious period in cattle was less than one day (27). However, other studies suggested that this phase may be substantially longer, potentially lasting several days (28, 29). Recent experiments demonstrated a distinct period of preclinical infectiousness in pigs and cattle (30, 31). These findings were consistent with earlier work that identified FMDV incubation phase transmission in piglets, lambs, calves, and dairy cattle and provided evidence that a defined preclinical infectious period should be explicitly included when modeling FMDV transmission in swine and cattle (29).

The quantitative impacts of FMDV incubation phase transmission in group-housed pigs have also been described (24). Modeled simulations of FMD outbreaks in U.S. swine found that the addition of a preclinical infectious period had significant impacts on the number of farms infected and pigs depopulated under suboptimal outbreak response (24). Specifically, a preclinical infectious period of one day resulted in a 40% increase in the median number of farms affected compared to a scenario with no incubation phase transmission (24). These results demonstrate the importance of explicitly including incubation phase transmission when modeling FMDV transmission in the U.S. pig production system.

Farm-to-farm transmission of FMDV occurs through multiple pathways, including direct animal contacts and indirect mechanisms, such as contaminated vehicles, personnel, fomites, and airborne spread. Animal transport significantly heightens transmission risks, as livestock frequently mix during movements for breeding, sale, slaughter, or finishing, increasing potential exposure events (32, 33). Containment efforts are further complicated by the occurrence of incubation phase transmission, highlighting the critical importance of enforcing movement restrictions once an outbreak is detected (5, 24).

Livestock congregation points, such as cattle markets, are prominent hotspots for virus dissemination, facilitating both direct and indirect transmission routes. Experimental evidence shows that viable FMDV can persist within contaminated environments, significantly extending the risk of transmission even without direct animal contact (34). Additionally, biosecurity lapses at farms increase this risk, as inadequate disinfection of vehicles, equipment, footwear, and clothing can mechanically transport the virus between premises (35, 36).

Given the complexity and interconnectedness of modern livestock systems, regional biosecurity strategies and coordinated surveillance efforts are essential to effective FMD control. Extensive commercial networks can quickly amplify disease outbreaks, especially when considering the risks associated with preclinical transmission (20, 37). For these reasons, effective outbreak management requires comprehensive biosecurity measures, stringent movement controls, and coordinated outbreak response. These activities should be guided by empirical data collected through transmission studies and epidemiologic modeling (24, 37). In the current study, these approaches demonstrate the relevance of considering transmission during the preclinical phase of infection when modeling or responding to outbreaks of FMD amongst cattle.

Epidemiologic models are relied upon for emergency preparedness in regions where historic outbreak data are limited. Since many of the challenges in FMD control are directly linked to strain-specific virus characteristics, transmission models must address these particular challenges (20). The reliability of such mathematical models depends upon a thorough understanding of disease dynamics and the elucidation of key epidemiologic parameters (20, 24, 31). Due to the great diversity of strain-specific FMD virus characteristics, it is imperative that parameters derived for use in epidemiologic models are representative of specific host-pathogen combinations (20, 31). To this end, transmission experiments carried out in high-containment research laboratories are a valuable source for deriving such parameters (24).

Traditionally, epidemiologic models of FMDV-spread have not included a distinct phase of preclinical transmission. While FMDV transmission during the incubation phase has been documented in cattle, pigs, and lambs, the impact of preclinical infectiousness and the value of including this phase in disease spread modeling have not been fully evaluated (24, 29, 31). The current investigation builds upon prior work conducted in swine (24) to quantify the impacts of FMDV incubation phase transmission on outbreak extent and epidemic duration in the U.S. cattle production system. The objectives of this study were to simulate FMDV transmission during the incubation phase in cattle, to evaluate the importance of including incubation phase transmission in epidemiological models, and to quantify the impacts of preclinical transmission when detection of the disease is optimal or suboptimal (as described in Methods Section 2.6).

## 2 Methods

### 2.1 Infectivity parameters

The current investigation integrated data collected through experimental studies performed by the Agricultural Research Service (ARS) of the U.S. Department of Agriculture (USDA) at the Plum Island Animal Disease Center (PIADC) investigating FMDV pathogenesis and infection dynamics in cattle (31). Yadav et al. (31) reported estimated durations of FMD disease phases for five distinct strains from three serotypes (O, A, and Asia 1) and all analyzed strains collectively (pan-serotypic). These parameters were found to depend upon several factors, including the effective exposure dose, route of exposure, and donor species. Additionally, there was variability between the viral strains examined. For example, strains within serotype A had a long subclinical infectious phase relative to serotypes O and Asia 1. Two other publications reported estimated durations of the preclinical phase that are comparable to those reported by Yadav et al. (31), Mardones et al. (38), and Cabezas et al. (39). In the current study, a series of incubation phase transmission models were developed using parameters that encompass the range variability appreciated between and within FMDV strains. These state-transition models were used to evaluate the impact of daily alterations in the duration of incubation phase transmission on simulated FMD outbreaks in the U.S. cattle production system.

### 2.2 Simulation modeling of FMD outbreaks in the United States

Simulations were performed using Interspread Plus CFE v6.2, a spatially explicit, stochastic simulation model of disease spread (40). The U.S. National FMD-Spread Model developed at the USDA Center for Epidemiology and Animal Health (CEAH) was reconfigured to assess the impacts of incubation phase transmission of FMD in cattle on simulated FMD outbreaks in specified regions of the country (41). For each regional scenario described below, FMDV was introduced on two index farms on simulation day 10. No infection or transmission events were simulated before simulation day 10. After disease introduction, farms transitioned through disease states in daily increments. Disease spread was simulated via animal movements, indirect contacts, local spread, and airborne spread. Transmission rates are included in the supporting materials. Regional farm population files were generated using U.S. Census of Agriculture data collected by the USDA, National Agricultural Statistics Service (NASS). The Farm Location and Agricultural Production Simulator (FLAPS) was used to simulate the cattle populations on individual facilities (42). After completing model validation to ensure that disease transmission in the model occurred during the expected phases of disease progression, we ran disease spread scenarios in multiple regions of the country.

### 2.3 Selection of index cases

Simulations were run for multiple starting locations throughout the U.S., with priority given to areas at heightened risk for FMDV introduction through commercial dairy farms. Locations identified at the highest risk for FMDV introduction through cattle of any class include the Pacific Coast, northeastern Texas, and Arkansas (43). Additionally, states with the largest number of licensed dairy herds, including Wisconsin, Pennsylvania, New York State, Minnesota, Ohio, and California, may be considered at an increased risk for an FMDV outbreak originating in dairy cattle (44). The ongoing H5N1 incident in U.S. dairy cattle also highlights the potential for infectious disease spread between dairy herds in these regions, as several of the states identified at a heightened risk for FMDV introduction have also reported cases of H5N1 in dairy cattle (45).

**Figure 2:**
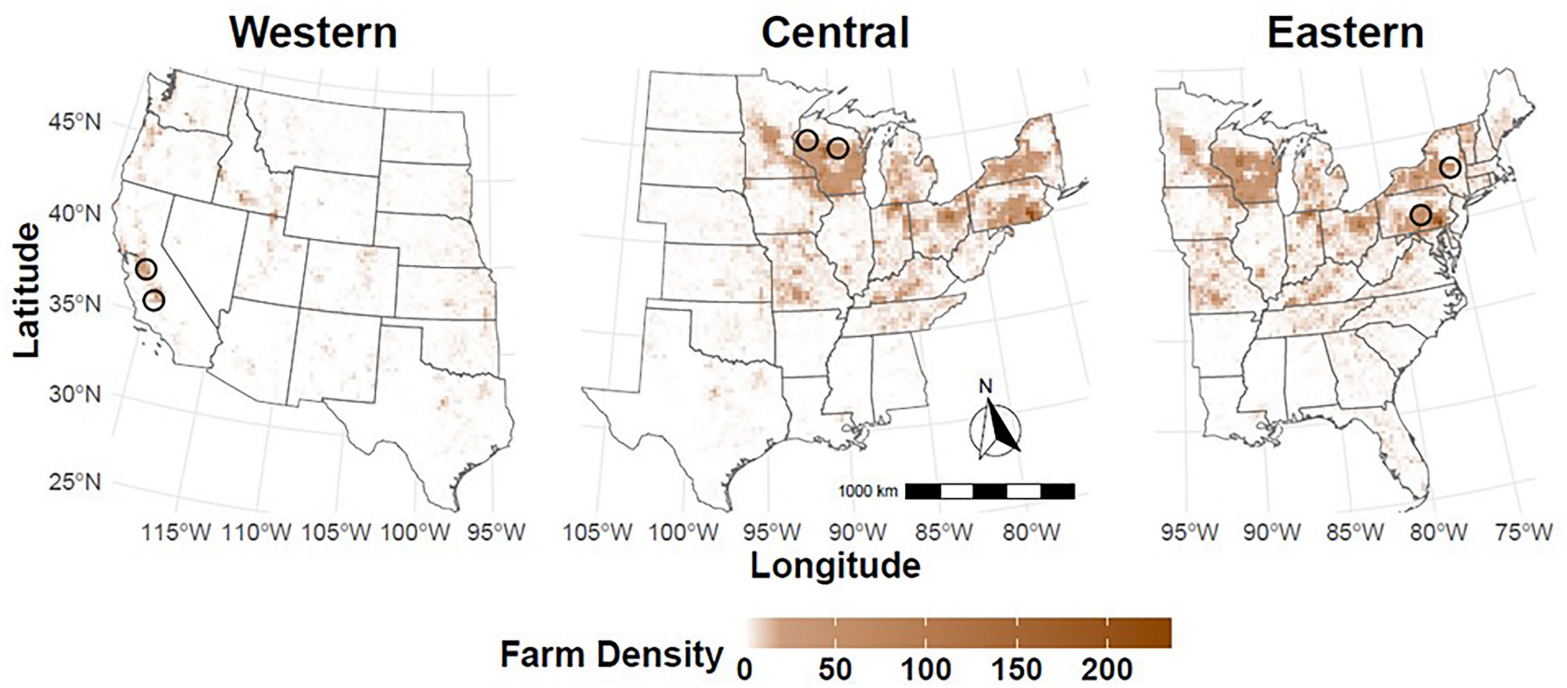
Regional scenario maps. FMDV outbreak simulations were seeded in dairy-dense areas in three regions of the country: Western U.S. (left), Central U.S. (middle), and Eastern U.S. (right). For each scenario, FMDV was introduced on two dairy farms on simulation day 10. Approximate locations for index farms are indicated with a black circle. Map shading represents the density of dairy farms.

States identified at a heightened risk for FMDV introduction through commercial dairy farms are located in multiple regions of the country with unique agricultural demographics and distributions of dairy operations. For this reason, outbreaks were seeded in dairy-dense areas in three regions (Figure 2). The conterminous U.S. was divided roughly in half geographically by east and west. Geographic regions, associated states, and the number of cattle farms are listed in Supplementary Table S1. In each region, two index farms were randomly selected from a subset of the model population file for the states chosen. The Western U.S. region included 391,632 cattle operations with 16 production types and a total of 55.6 million cattle across 17 states. To simulate FMDV-spread throughout the Western U.S., FMDV introduction was simulated on two large, commercial dairies located in California on simulation day 10.

To simulate spread throughout the Eastern U.S., FMDV introduction was simulated on one large, commercial dairy in New York state and a second large, commercial dairy in Pennsylvania. Both farms were infected on simulation day 10. Pennsylvania and New York are among the top 3 states by number of licensed dairy herds (44). While small dairies are predominant in this region, simulating FMDV introduction on large dairy farms resulted in more consistent disease spread and facilitated comparison between regional scenarios (44). Simulations were run using a regional farm file composed of 498,127 cattle operations with 15 production types and a total of 32.4 million cattle across 31 states in the Eastern U.S. There were no large cattle feedlot premises located within the Eastern U.S. FMD farm file. All 48 states of the conterminous U.S. were represented in the Western and Eastern U.S. regional scenarios, together including 88.0 million cattle on 889,759 cattle premises.

A third regional scenario was developed to account for spread eastward and westward from the Central U.S. FMDV introduction was simulated on two large, commercial dairies located in Wisconsin, the state with the greatest number of licensed dairy herds nationally (44). As in the Western U.S. and Eastern U.S. scenarios, index farms were infected on simulation day 10. Simulations were run using a regional farm file composed of 673,317 cattle operations with 16 production types and a total of 59.5 million cattle. Of the 23 states included in the Central U.S. regional scenarios, 6 states were represented in the Western U.S. scenarios and 17 in the Eastern U.S. scenarios.

### 2.4 Modeling infection dynamics

**Figure 3:**
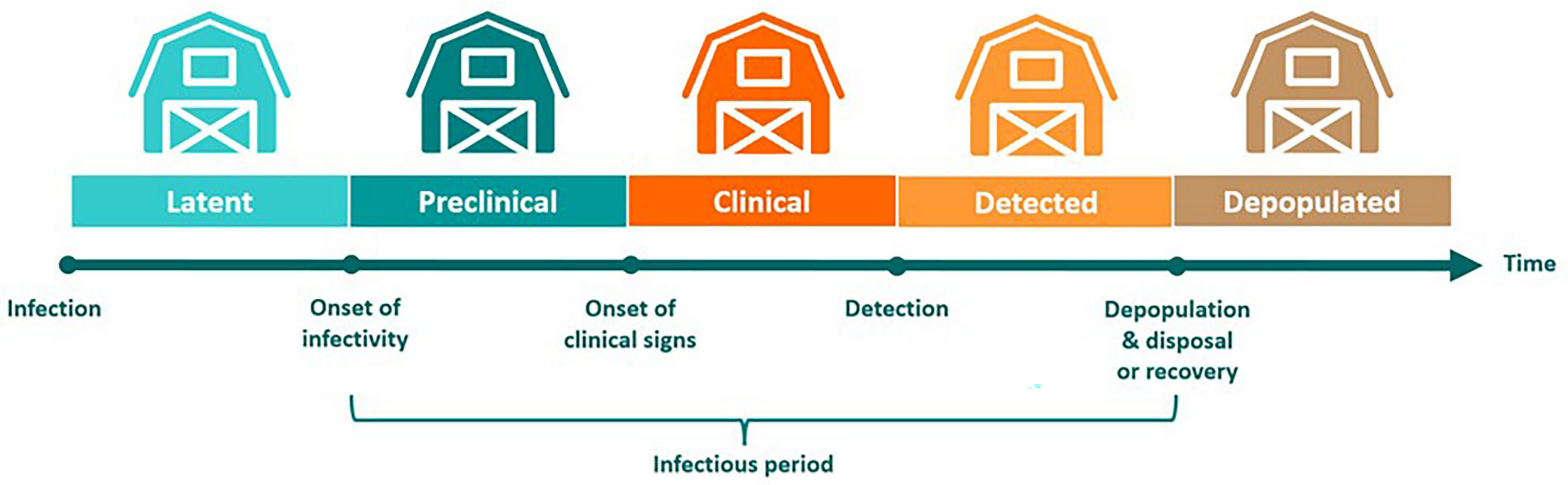
Modeling distinct phases during an outbreak of foot-and-mouth disease. Following exposure to infectious FMDV, infected farms transitioned through several stages of disease that reflect those taking place in individual animals. Since our model uses the farm as the infectious unit, all cattle on a premises are considered infected once a single animal has contracted the virus. Farms transition through latent, preclinical infectious, clinical infectious, detected, and depopulated states. On large cattle feedlots with >35,000 cattle, animals are allowed to recover-in-place.

After FMDV exposure, infected farms transitioned through latent, preclinical infectious, clinical infectious, detected, and depopulated states (Figure 3). Affected farms remained infectious from onset of infectivity until completion of carcass disposal following depopulation. On large cattle feedlots with >35,000 animals, where depopulation and disposal of carcasses in an effective and timely manner is not feasible, cattle were exempt from depopulation (46). Cattle on these operations were allowed to recover-in-place while movement restrictions were enforced, which has been described as an effective alternative when mass depopulation is not practical (47). Since our model uses the premises as the infectious unit, once a farm became infected, all cattle on the farm were assumed to be infected and therefore subject to depopulation or recovery-in-place.

To simulate FMDV transmission during the incubation phase, the preclinical infectious duration was altered in daily increments of 1, 2, and 3 days. These values were chosen to encompass the range of estimated preclinical infectious durations reported for a diversity of FMDV strains in the literature and those derived from experimental studies conducted at the Plum Island Animal Disease Center (26, 38, 39). Model simulations including a distinct period of incubation phase transmission were compared to baseline scenarios with no preclinical transmission. For all scenarios, infected farms had a constant incubation phase of 4 days, such that the latent phase was shortened by 1 day with each additional day of incubation phase transmission. All baseline and incubation phase transmission models were run for 500 iterations using the same seed.

A reparameterization of the incubation phase transmission model described above was developed to simulate spread of a FMDV strain with low virulence in cattle, such as strain A/SKR/2010 isolated in 2010 from an outbreak in the Republic of Korea (48). The purpose of this variation was to investigate the impacts surrounding a biologically slower virus that would be more difficult to detect due to low-grade clinical signs, as is known to exist within the spectrum of FMDV strains. For the low-virulence FMDV scenarios, a 4-day latent period, 6-day preclinical infectious phase, and a total incubation period of 10 days were simulated with suboptimal detection. The low-virulence FMDV model was run for 450 iterations in the Central U.S. and for 500 iterations in the Western and Eastern U.S. All simulations were run for one year.

### 2.5 Outbreak response conditions and control measures

For each scenario, the extent and duration of simulated outbreaks were evaluated when detection of the disease was optimal and when detection was suboptimal. Specifically, the time between the onset of clinical signs and the first detection were varied, as well as the rate of detection of subsequent cases. Under optimal response conditions, detection of the index case occurred 1 day after the onset of clinical signs (5 days post infection). Initial detection in the model occurred on simulation day 15. Under suboptimal response conditions, the first detection was postponed until 4 days after clinical onset corresponding to 8 days post infection (simulation day 18). This delay accounts for producer observation of clinical disease (passive surveillance), sample collection by a Foreign Animal Disease Diagnostician (FADD), confirmatory diagnostic testing at a national laboratory, and disease reporting (5). The suboptimal detection scenarios also included a short delay in detection of subsequent cases via passive and active surveillance. The low-virulence FMDV scenarios utilized suboptimal response conditions with initial detection occurring 4 days after clinical onset or 14 days post infection (simulation day 24).

**Figure 4:**
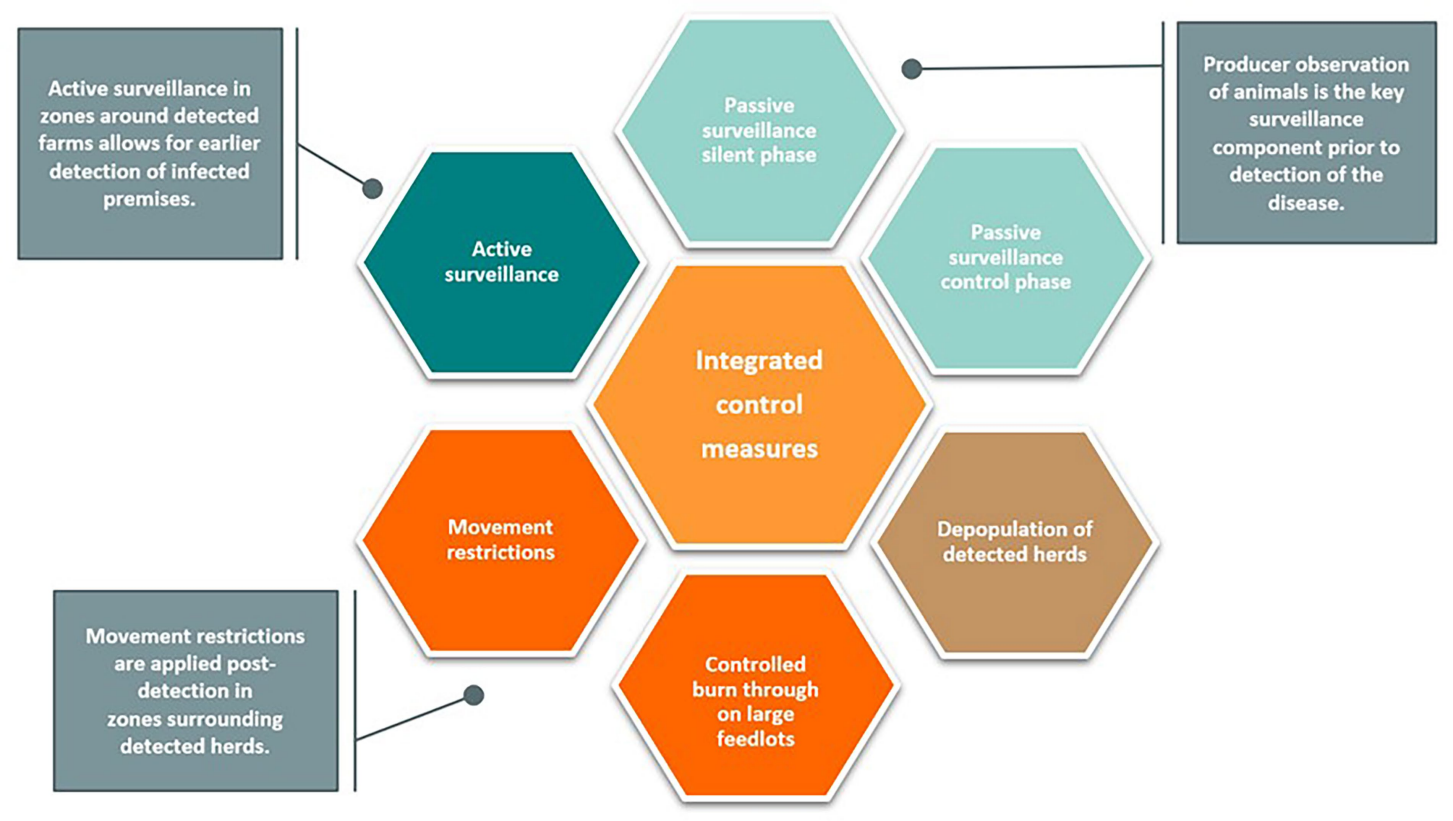
Integrated control measures. Several response measures are initiated during an FMD outbreak. Initial detection of the disease relies upon passive surveillance by producers. Once an outbreak is identified, active surveillance allows for earlier detection of subsequent cases. Additional control measures include movement restrictions, depopulation of detected herds, and controlled burn through (recovery-in-place).

Integrated control measures, illustrated in Figure 4, were initiated upon detection of the index cases. The response measures modeled reflect recommendations outlined in the U.S. FMD Response Plan (5). Movement restrictions were implemented in 0–10 km Control Zones around detected premises and were consistent across all scenarios. Active surveillance was conducted at cattle markets, processors, and within 0–10 km Control Zones and 10–20 km Surveillance Zones. These active surveillance strategies allowed for the detection of farms during the preclinical infectious phase, before the onset of clinical FMD (Supplementary Table S3). Farms eligible for depopulation were processed at a rate determined by the farm class, herd size, and resource availability. These parameters were derived from expert opinion and were consistent between all modeling scenarios evaluated.

### 2.6 Statistical analysis

Statistical analyses were conducted in R to assess the impacts of preclinical transmission on simulated outbreak outcomes under two detection scenarios (optimal versus suboptimal) and for various durations of incubation phase transmission (0 to 3 days). Estimates for the magnitude and duration of simulated outbreaks were used to determine the impacts of these parameters. To assess outbreak size and magnitude, the number of infected cattle was calculated as the total number of cattle on infected premises. The cumulative spatial spread was also calculated as the area under the curve (AUC) for daily transmission distances. These were compared between scenarios. Cumulative case detections were also plotted to assess geographic extent. To evaluate the epidemic duration of simulated outbreaks, the number of days between the first and last detection of a single outbreak was calculated and compared between scenarios.

Simulated FMDV outbreaks were further characterized using multiple epidemiological outcome variables, including the effective reproduction number (Re) and epidemic velocity. To calculate daily Re, the number of transmission events from each source farm were counted on every day of the simulation. The median daily Re was calculated across all iterations and used to estimate epidemic potential. The epidemic velocity (km/day) was calculated as the average distance between each source and destination farm for each day of the simulation. Epidemic velocity was used to assess the timing of peak transmission, where peak day represents the simulation day with the greatest daily spread (km). To quantify the median cumulative spatial spread of disease, we calculated the area under the curve (AUC) of the daily transmission distance over the entire outbreak period for each simulation iteration. A linear regression analysis was conducted on the logarithm of the AUC of daily spread rates to evaluate the influence of preclinical infectious duration and response strategy on the cumulative spread in each region.

Due to skewness, outcomes representing counts and cumulative metrics, such as the number of infected premises, total cattle infected, cumulative spatial spread, and daily distance, were log-transformed using the logarithm. For all outcome variables, ordinary least squares (OLS) regression models were used, modeling each as a function of the preclinical infectious duration and detection scenario (optimal versus suboptimal), including interaction terms to assess whether responsive effectiveness varied with infectious duration. The statistical significance of individual predictors was determined using t-tests. The overall model significance was assessed using F-tests. The same methodological approach was applied consistently for each of the Western, Eastern, and Central regional scenarios.

## 3 Results

**Figure 5:**
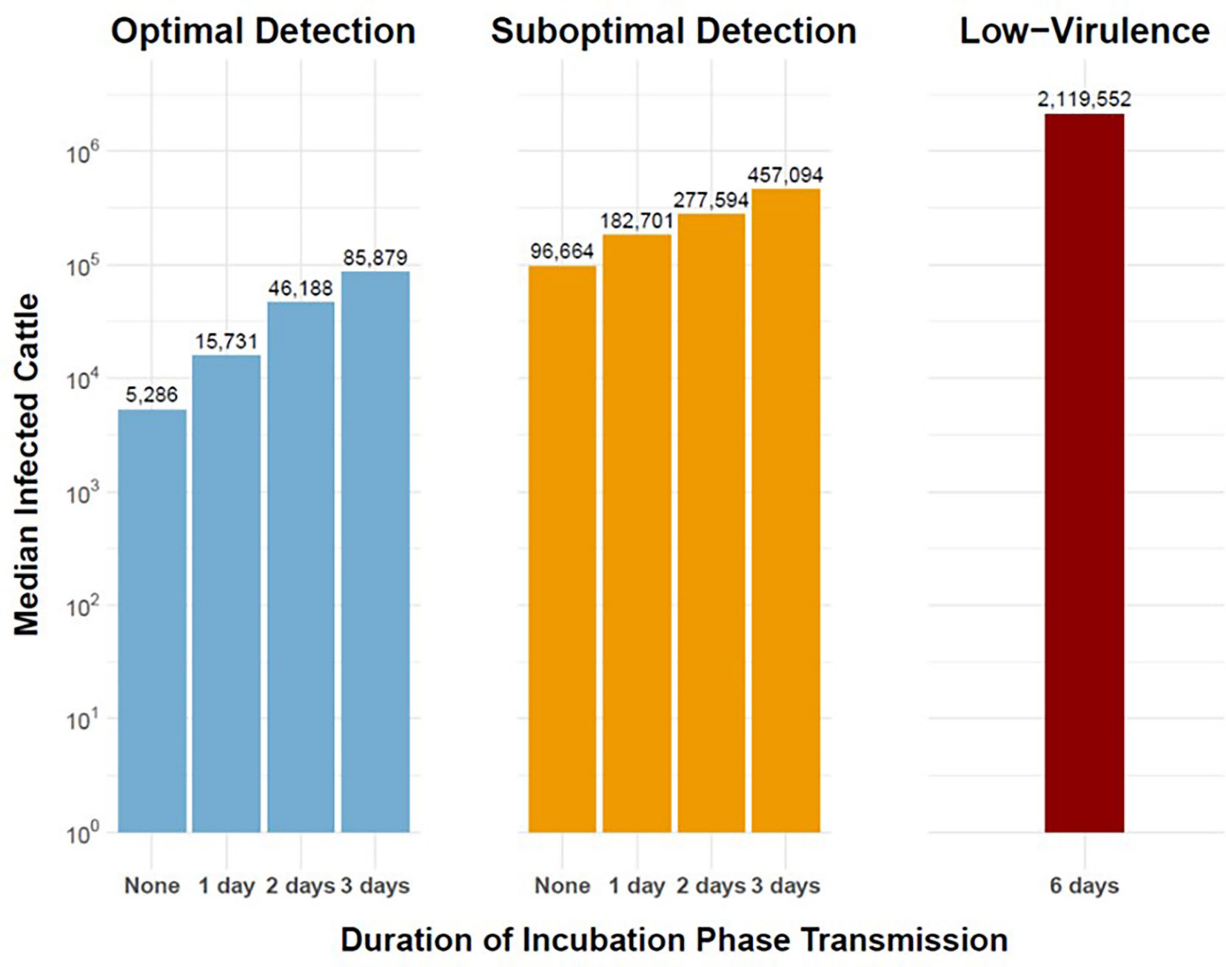
Magnitude of simulated outbreaks of FMD in cattle in the Western U.S considering variable duration of preclinical infectiousness. Vertical bars depict median number of infected cattle on a logarithmic scale (vertical axis) for varying durations of incubation phase transmission (horizontal axis). Panel columns indicate detection scenarios: Optimal Detection (left), Suboptimal Detection (middle), and Low-Virulence FMDV (right). Workflow of the analyses and figures for the Eastern U.S. and Central U.S. are provided as Supplementary materials and are available at: https://geoepi.github.io/FMD-preclinical-spread/.

**Figure 6:**
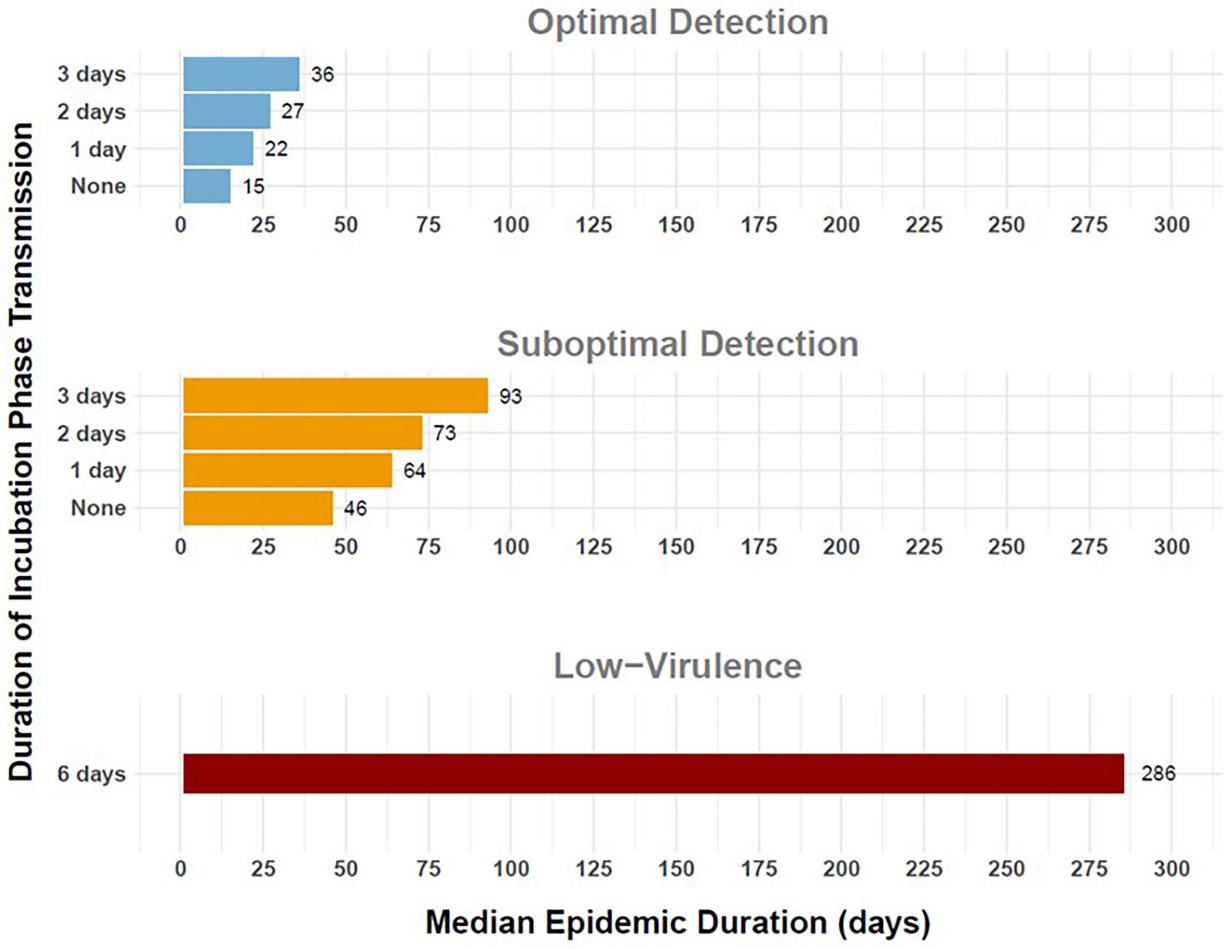
Duration of simulated outbreaks of FMD in cattle in the Western U.S considering variable duration of preclinical infectiousness. Horizontal bars depict median epidemic duration in days (horizontal axis) for varying durations of preclinical infectiousness (vertical axis). Panel rows indicate detection scenarios: Optimal Detection (top), Suboptimal Detection (middle), and Low-Virulence FMDV (bottom). Workflow of the analyses and figures for the Eastern U.S. and Central U.S. are provided as Supplementary materials and are available at: https://geoepi.github.io/FMD-preclinical-spread/.

**Figure 7:**
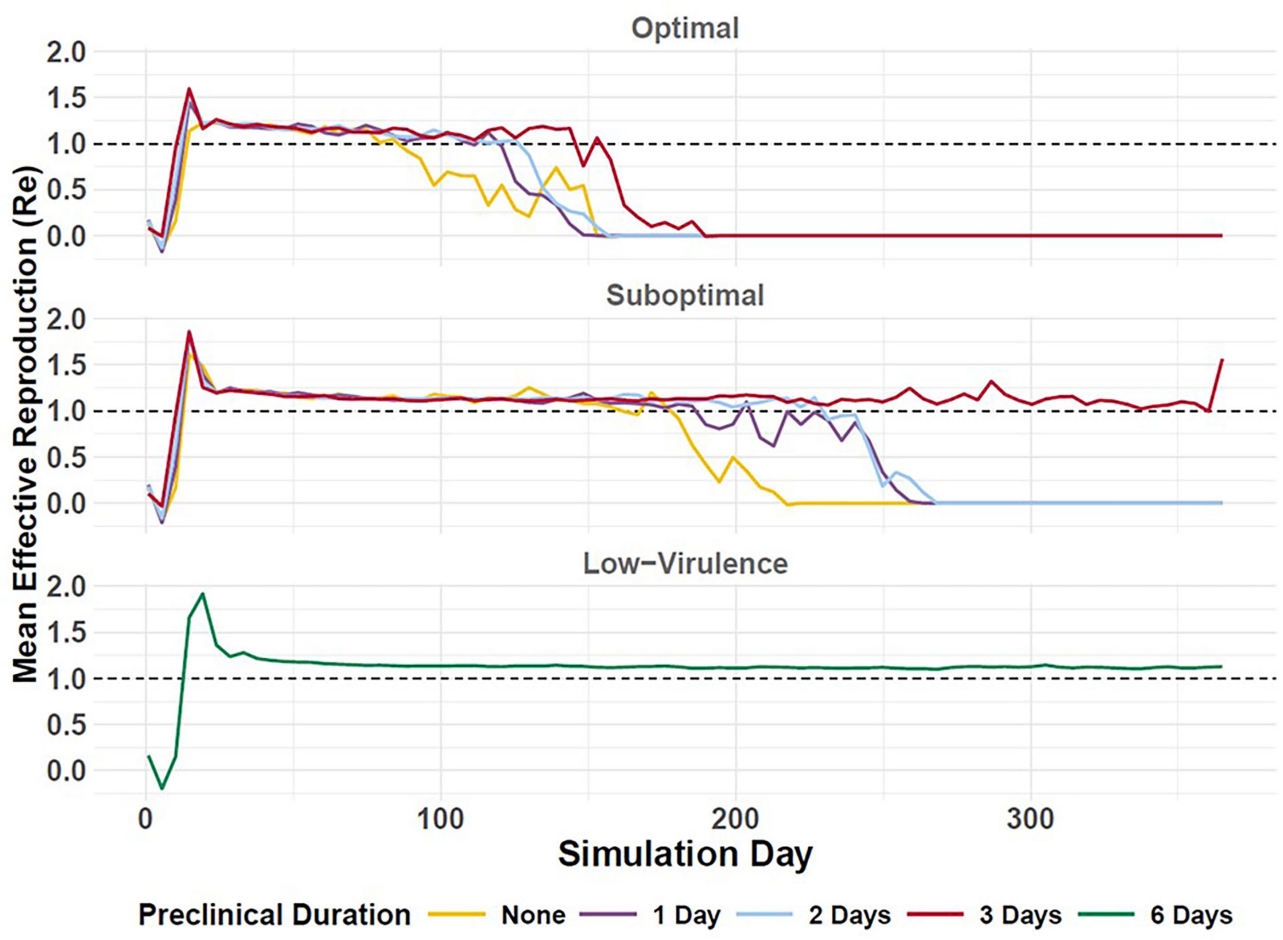
Effective reproduction (Re) of simulated FMD outbreaks in cattle in the Western U.S. Vertical axis depicts Re (mean) in daily increments (horizontal axis). Durations of preclinical infectiousness are color-coded according to legend at bottom with detection scenarios indicated by panel. Workflow of the analyses and figures for the Eastern U.S. and Central U.S. are provided as Supplementary materials and are available at: https://geoepi.github.io/FMD-preclinical-spread/.

**Figure 8:**
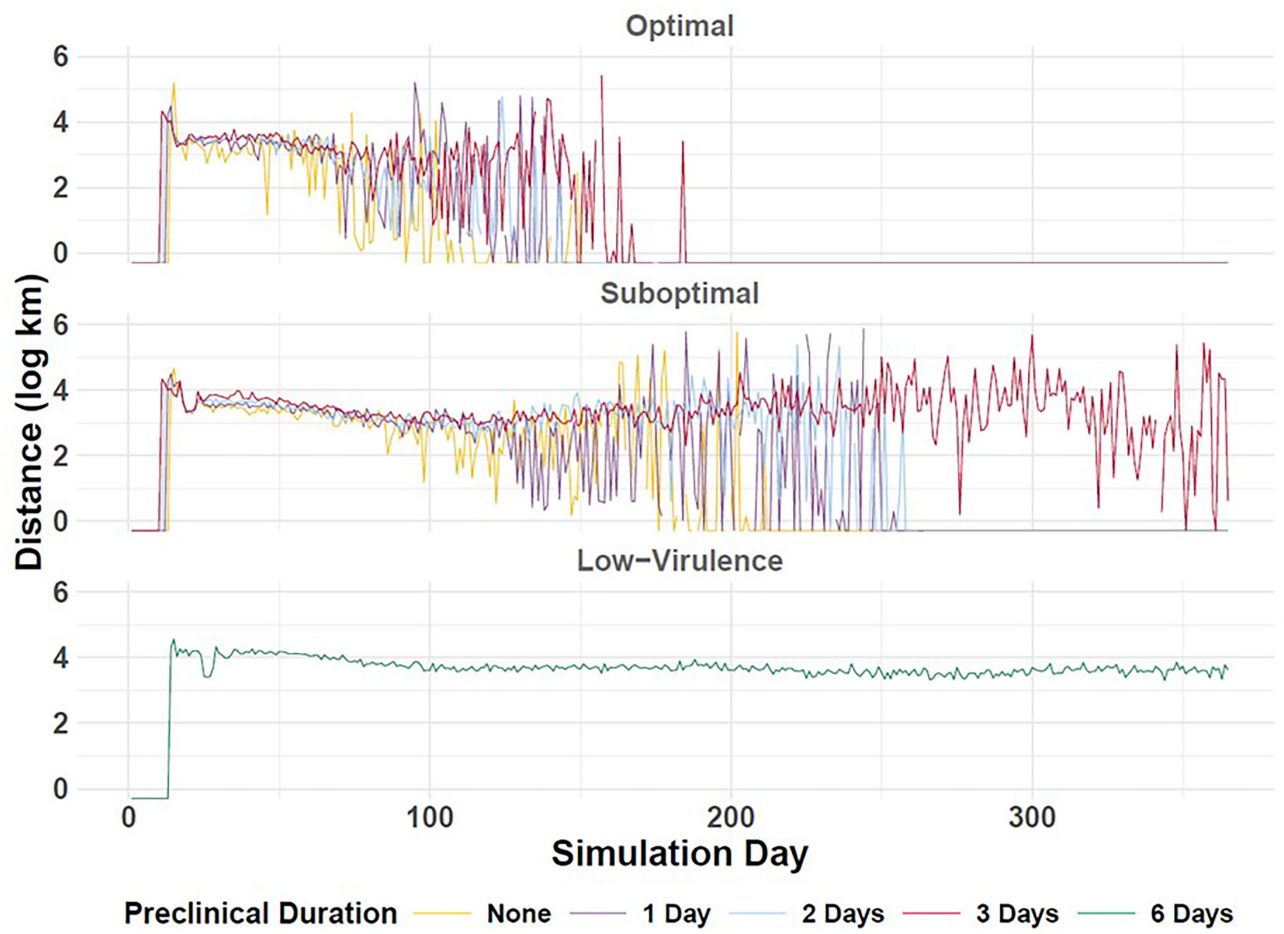
Epidemic velocity of simulated FMD outbreaks in the Western U.S. Vertical axis depicts outbreak geographic spread distance (log km) in daily increments (horizontal axis). Durations of preclinical infectiousness are color-coded according to legend at bottom with detection scenarios indicated by panel. The occurrence of peak transmission corresponds to the simulation day on which distance is longest. Workflow of the analyses and figures for the Eastern U.S. and Central U.S. are provided as Supplementary materials and are available at: https://geoepi.github.io/FMD-preclinical-spread/.

**Figure 9:**
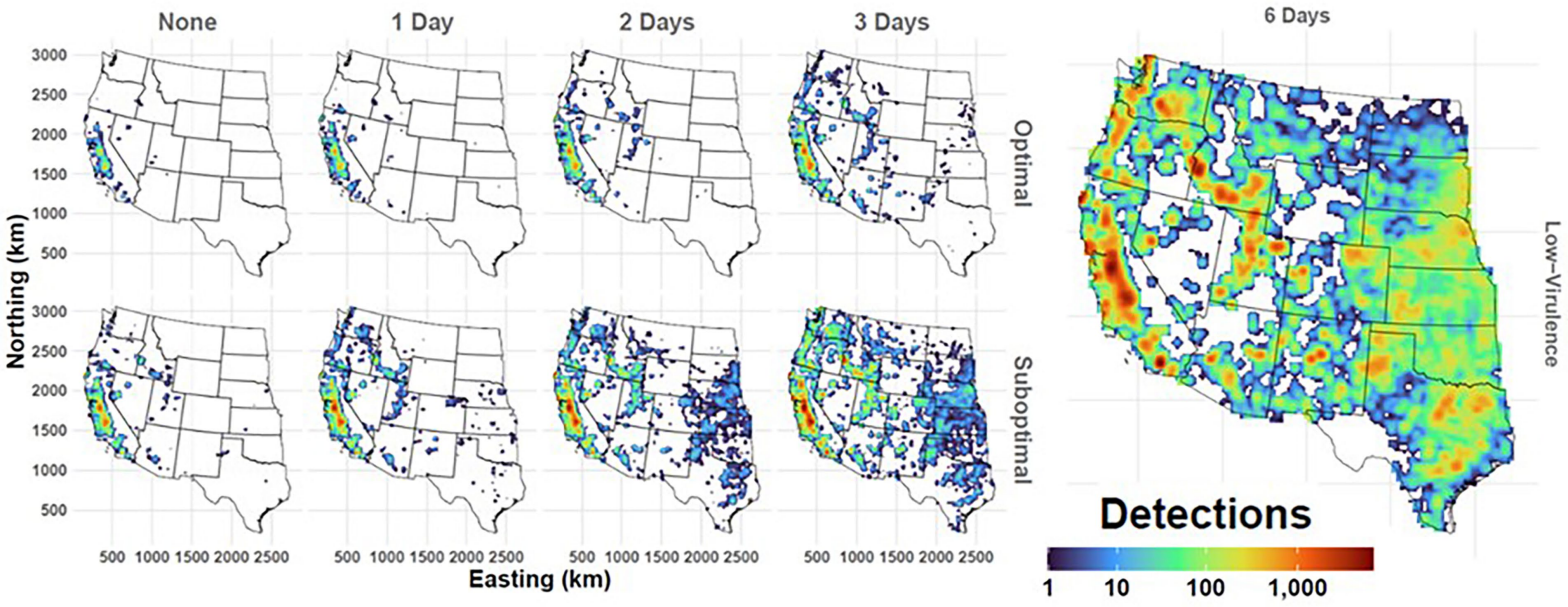
Geographic extent of simulated FMD outbreaks in the Western U.S. Maps depict cumulated case detections as aggregated to a 25x25km grid. Panel columns indicate duration of the preclinical infectious phase with rows showing detection scenario. Legend at bottom right reports the cumulative number of case detections per grid cell resulting from 500 simulated outbreak iterations. Workflow of the analyses and figures for the Eastern U.S. and Central U.S. are provided as Supplementary materials and are available at: https://geoepi.github.io/FMD-preclinical-spread/.

In all three geographic regions evaluated, the duration of preclinical (incubation) phase transmission was a determinant of FMDV-spread, outbreak extent, and epidemic duration (Figures 5–9). Scenarios with no incubation phase transmission consistently contained fewer farms and shorter outbreak durations when compared to scenarios that included a preclinical infectious phase. Each additional day of incubation phase transmission resulted in a greater median number of cattle infected and a longer median outbreak duration under both optimal and suboptimal detection. Overall, the impacts of incubation phase transmission were more profound when detection of index and subsequent cases were suboptimal than when detection was optimal. This underscores the critical importance of rapid disease detection and response efforts. While these trends were conserved across regions, there were regional differences in the interaction effects between response strategy and preclinical infectiousness.

### 3.1 Magnitude of simulated outbreaks

To assess the impacts of incubation phase transmission on the magnitude of simulated FMD outbreaks, a linear regression analysis was conducted on the logarithm of total cattle infections. In all three regions, increasing preclinical infectious duration was significantly associated with larger outbreaks, with estimated increases in infected cattle for each day of increase in preclinical infectiousness (*p* < 0.001 at all levels). Although an optimal response was also associated with a reduction in outbreak size in each region (all *p* < 0.001), the significance of interaction terms between preclinical phase duration and response strategy varied regionally. Of all model simulations evaluated, the low-virulence FMDV scenario in the Central U.S. resulted in the greatest outbreak size with a median of 3.4 million cattle infected with FMDV during the first year of the outbreak (Figure 5).

Simulated FMDV-spread in the Western U.S. indicated that under suboptimal detection, the addition of a single day of incubation phase transmission resulted in an 89% increase in the median number of cattle on infected premises from 96,664 cattle in the baseline scenario to 182,701 cattle in the scenario with a 1-day preclinical infectious phase (Figure 5). Additionally, the low-virulence FMDV scenario resulted in larger outbreaks with a median of 2.1 million cattle infected with FMDV during the first year. An optimal response was associated with a substantial reduction in outbreak size (*β* = −1.78, *p* < 2e–16). Notably, the interaction terms between preclinical phase duration and response strategy were not statistically significant (all *p* > 0.05), indicating that the mitigating effect of an optimal response did not vary across duration levels in the Western region. The overall model was highly significant [*F*(7, 3,992) = 371.3, *p* < 2.2 × 10^–16^] and accounted for approximately 39% of the variance in log-transformed cattle infections. These results suggest that, while prolonged preclinical duration infectiousness markedly increases outbreak magnitude, the benefit of an optimal response is consistent regardless of phase duration in the Western region.

In the Eastern U.S., increasing preclinical infectious duration was significantly associated with larger outbreaks (*p* < 0.001 at all levels) and an optimal response was associated with a reduction in outbreak size (*β* = −1.40, *p* < 2e–16). Significant interaction effects were observed for 2 day (β = −0.31, *p* = 0.009) and 3 day (β = −0.66, *p* < 2.21 × 10–08) preclinical infectious periods, indicating that the mitigating effect of an optimal response became more pronounced as preclinical phase duration increased. By comparison, the interaction with only a 1-day preclinical phase was not statistically significant. The overall model was highly significant [*F*(7, 3,992) = 348.6, *p* < 2.2 × 10–16] and accounted for approximately 38% of the variance in log-transformed cattle infections in the Eastern region.

For the Central region, increasing preclinical infectious duration was significantly associated with larger outbreaks (*p* < 0.001 at all levels) and an optimal response was associated with a substantial reduction in outbreak size (*β* = −1.53, *p* < 0.001). In contrast to the Western region, in the Central region, the mitigating effect of an optimal response became more pronounced as the duration of the preclinical phase increased (β = −0.28, −0.56, and −0.84 for 1, 2, and 3 day durations, respectively; all *p* < 0.001). The overall model was highly significant [*F*(7, 3,992) = 385.3, *p* < 2.2 × 10^–16^] and accounted for approximately 40% of the variance in log-transformed cattle infections. These results suggest that both the preclinical infectious phase duration and the efficiency of FMDV detection influence outbreak dynamics.

### 3.2 Duration of simulated outbreaks

The duration of the FMD epidemic, calculated as the time between the initial detection and last detection of a single outbreak, was the longest for the low-virulence FMDV scenarios, with over 50% of simulated outbreaks exceeding 9 months, 10 months, and 11 months in the Western, Eastern, and Central regional scenarios, respectively. The shortest outbreak duration occurred in the scenarios with no incubation phase transmission for all three geographic regions and both response categories. A linear regression analysis was conducted to evaluate the effect of preclinical infectious duration and response strategy on epidemic duration in each region.

In the Western region, the addition of a single day of incubation phase transmission resulted in an 18-day increase in the median epidemic duration from 46 to 64 days when detection was suboptimal (Figure 6). Longer preclinical infectious periods were significantly associated with extended outbreaks, with increases in average epidemic duration of 15.18, 28.07, and 47.25 days for 1, 2, and 3 day durations, respectively (all *p* < 0.001). An optimal response was also associated with reduced outbreak duration (*β* = −32.39, *p* < 2 × 10^–16^). Furthermore, significant interaction effects indicated that the impact of longer preclinical infectious periods on outbreak duration was partially mitigated under an optimal response, with interaction estimates of −7.70, −11.50, and −24.00 days for 1, 2, and 3 day durations, respectively (all *p* < 0.05). The overall model was highly significant [*F*(7, 3,992) = 290.1, *p* < 2.2 × 10^–16^] and explained approximately 33.7% of the variance in outbreak duration in the Western U.S.

Simulated FMDV-spread in the Eastern U.S. indicated that each additional day of preclinical infectiousness was associated with increased outbreak duration (all *p* < 0.001) and an optimal response was associated with an estimated reduction in outbreak duration of 30.49 days compared to a suboptimal detection (*p* < 2 × 10^–16^). The effect of prolonged preclinical duration on extending outbreak length was attenuated under an optimal response, with significant interaction terms at 2 day (*β* = −18.32) and 3 day (β = −37.14) preclinical infectious periods. The overall model was highly significant [*F*(7, 3,992) = 253.2, *p* < 2.2 × 10^–16^] and explained approximately 31% of the variance in outbreak duration in the Eastern U.S.

Consistent with the Western and Eastern regions, each additional day of preclinical infectiousness was associated with increased outbreak duration in the Central U.S. (*p* < 0.001). An optimal response was associated with a substantial reduction in duration when compared to a suboptimal response (β = −32.66, *p* < 2 × 10^–16^). Our results indicate that the effect of prolonged preclinical infectiousness on outbreak duration was attenuated under an optimal detection in the Central U.S. as well. The overall model was highly significant [*F*(7, 3,992) = 295.3, *p* < 2.2 × 10^–16^] and accounted for approximately 34% of the variance in outbreak duration observed in the Central U.S.

### 3.3 Effective reproduction (Re)

To assess variation in FMD outbreak intensity, we estimated the farm-level median effective reproduction number (Re), which reflects the number of secondary farms infected by a primary farm. We applied ordinary least squares regression to the average Re to evaluate the influence of preclinical infectious phase duration and response strategy on epidemic potential in each region. Overall, longer durations of incubation phase transmission were associated with higher epidemic potential, as measured by increased Re.

In the Western U.S., longer durations of preclinical transmission were associated with higher Re when detection was suboptimal (Figure 7), with estimated increases of 0.13, 0.18, and 0.54 for preclinical phase durations of 1, 2, and 3 days, respectively (all *p* < 0.001). An optimal response was associated with a significant reduction in average Re (*β* = −0.24, *p* < 2 × 10^–9^). The interaction between preclinical duration and response strategy was significant for preclinical infectious durations of 2 (β = −0.11, *p* = 0.045) and 3 days (β = −0.40, *p* < 2.9 × 10^–12^), suggesting that the mitigating effect of an optimal response becomes more pronounced as the preclinical phase lengthens; however, the interaction for a preclinical phase duration of 1 day was marginally non-significant (*p* = 0.097). The overall model was highly significant [*F*(7, 2,912) = 84.21, *p* < 2.2 × 10^–16^] and accounted for approximately 16.8% of the variance in Re.

When detection was suboptimal, increases in preclinical duration were significantly associated with higher Re in the Eastern region, with estimated increases of 0.20, 0.42, and 0.45 for preclinical infectious durations of 1, 2, and 3 days, respectively (all *p* < 0.001). The optimal detection scenario was associated with a reduction in Re of 0.37 compared to the suboptimal detection scenario (*p* < 2 × 10^–16^). Although the interactions for preclinical phase durations of 1 and 2 days were not statistically significant, the interaction for an infectious duration of 3 days was significant (*β* = −0.24, *p* = 2.16 × 10^–5^), indicating that the increase in Re with prolonged preclinical infectiousness is attenuated under the optimal response when infectious periods are longest. The overall model was highly significant [*F*(7, 2,912) = 113.9, *p* < 2.2 × 10^–16^] and explained approximately 21% of the Re.

Simulated FMDV-spread in the Central U.S. indicated that longer preclinical phase durations were significantly associated with higher epidemic potential as measured by increased Re, with estimated increases of 0.23, 0.29, and 0.58 for phase increases of 1, 2, and 3 days, respectively (all *p* < 0.001). An optimal response was associated with a significant reduction in Re (*β* = −0.20, *p* = 1.22 × 10^–6^). Interaction terms for preclinical infectious duration were statistically significant at 1 day (β = −0.26, *p* = 8.71 × 10^–6^) and 3 days (β = −0.26, *p* = 7.76 × 10^–6^), indicating that the Re was attenuated under an optimal response. The overall model was highly significant [*F*(7, 2,912) = 83.65, *p* < 2.2 × 10^–16^] and explained approximately 16.5% of the variance in log-transformed average Re in the Central U.S.

### 3.4 Epidemic velocity

To assess the timing of peak transmission of FMD, we performed an ordinary least squares regression for each region. The day of peak transmission was modeled as a function of preclinical infectious phase duration and response strategy, including an interaction term between these predictors. In general, longer preclinical infectious periods were associated with a later peak of transmission. When detection was suboptimal, there was an additional delay in peak spread observed in each regional scenario.

In the Western region, longer preclinical durations were significantly associated with a later occurrence of peak transmission, with an estimated increase of 4.63 days for each additional day of preclinical infectiousness (*p* < 2 × 10^–16^; Figure 8). The suboptimal response was also associated with an 8.60-day delay in peak spread relative to the optimal scenario (*p* < 2 × 10^–16^). A significant interaction between preclinical duration and response type (*β* = 3.26, *p* < 2 × 10^–16^) indicates that the effect of preclinical phase duration on peak transmission is amplified under the suboptimal response, further delaying peak transmission. The overall model was highly significant [*F*(3, 3,777) = 234.7, *p* < 2.2 × 10^–16^] and explained approximately 16% of the variance in epidemic velocity in the Western U.S.

As appreciated in the Western region, longer preclinical durations were significantly associated with a later peak, with an estimated delay of 4.71 days per unit increase in preclinical duration in the Eastern U.S. (*p* < 2 × 10^–16^). Moreover, a suboptimal response was associated with an additional delay of 6.52 days compared to the optimal scenario (*p* < 2 × 10^–16^). A significant interaction between preclinical duration and response type (*β* = 4.64, *p* < 2 × 10^–16^) indicated that the effect of prolonging the preclinical phase was further exacerbated under suboptimal response conditions. The overall model was highly significant [*F*(3, 3,709) = 178.7, *p* < 2.2 × 10^–16^] and explained approximately 12.6% of the variance in peak transmission timing in the Eastern region.

Longer preclinical durations were associated with a later day of peak transmission in the Central U.S. as well, with an estimated increase of 7.56 days per additional day of preclinical infectiousness (*p* < 2 × 10^–16^). The suboptimal response was associated with an extra delay of 7.15 days compared to the optimal response (*p* = 6.69 × 10^–7^). Furthermore, the effect of extended preclinical duration on delaying peak transmission was further amplified under the suboptimal response scenario (β = 6.91, *p* < 2 × 10^–16^). The overall model was highly significant (*F*(3, 3,692) = 251.8, *p* < 2.2 × 10^–16^) and explained approximately 17% of the variance in peak transmission timing in the Central U.S.

### 3.5 Cumulative spatial spread and geographic extent

In all three regions, increasing preclinical infectious duration was significantly associated with a larger cumulative spread (all *p* < 2 × 10^–16^) and greater geographic extent. A density map depicting cumulative case detections in the Western U.S demonstrates the impacts of incubation phase transmission and detection efficiency on the geographic extent of simulated outbreaks (Figure 9).

In the Western region, increasing preclinical infectious duration was significantly associated with a larger cumulative spread (*p* < 2 × 10^–16^) and greater geographic extent (Figure 9). A suboptimal detection resulted in a significant spread increase over an optimal detection (*p* < 2 × 10^–16^). Additionally, a significant interaction between preclinical phase duration and response type (*β* = 22.17, *p* < 2 × 10^–16^) indicated that the effect of infectious duration was further amplified under the suboptimal response. The overall model was highly significant [*F*(3, 3,500) = 494.2, *p* < 2.2 × 10^–16^] and accounted for approximately 30% of the variance in log-transformed cumulative spread. Furthermore, the low-virulence FMDV scenario resulted in simulated outbreaks of greatest magnitude and extent across the Western U.S. (Figure 9).

For the Eastern region, increasing preclinical phase duration was significantly associated with larger outbreaks (*p* < 2e-16). Detection scenario also significantly influenced cumulative spread, with significant interaction between preclinical phase duration and response type (*β* = 32.89, *p* < 2e-16), indicating that the mitigating effect of optimal response intensified as the preclinical duration increased. The overall regression model was highly significant [*F*(3, 3,429) = 409.3, *p* < 2.2 × 10^–16^] and accounted for approximately 26% of the variance in log-transformed cumulative spread in the Eastern region.

The same pattern as observed for the Western and Eastern regional scenarios was appreciated in the Central U.S. Increasing preclinical phase duration was significantly associated with larger cumulative spread, with geographic spread increases for each additional day of preclinical infectiousness (all *p* < 2 × 10^–16^). Suboptimal detection scenarios exhibited a significantly greater spread than the optimal detection scenarios (*p* < 2 × 10^–16^). A significant interaction between preclinical phase duration and response scenario type (β = 42.51, *p* < 2 × 10^–16^) indicated that increasing preclinical duration proportionally amplified spread in the suboptimal response scenario. The overall model was highly significant [*F*(3, 3,391) = 470.8, *p* < 2.2 × 10^–16^] and explained approximately 29% of the variance in log-transformed cumulative spread in the Central U.S.

## 4 Discussion

This investigation addresses the critical relevance of preclinical (incubation) phase transmission in determining the outcomes of simulated FMD outbreaks in the U.S. cattle production system. Our results demonstrate that the inclusion of even a single day of preclinical infectiousness exacerbates outbreak extent and duration under both optimal and suboptimal detection scenarios. These findings align closely with previous studies that emphasize the epidemiological importance of the incubation period in pigs and cattle (19, 33).

There are several important limitations to the current study that may influence interpretation and generalization of the results. For example, this study did not evaluate the influences of varying contact rates or movement patterns, although these factors are expected to influence outbreak dynamics. Additionally, the time taken to complete depopulation and disposal of large cattle operations may result in a prolonged infectious period, further contributing to disease spread. This concern highlights the roles of biosecurity in reducing local spread from detected and depopulated farms, and active surveillance in detecting new cases earlier in disease progression. Enforcement of movement restrictions on detected premises and those within control zones is also essential to mitigate this risk.

### 4.1 Regional patterns

Our analyses demonstrated consistent regional patterns across epidemiological metrics, highlighting the critical role of preclinical infectious phase duration and response effectiveness in shaping FMD outbreak dynamics. In all regions analyzed, longer preclinical infectious durations significantly increased outbreak magnitude, cumulative spatial spread, epidemic velocity, Re, and outbreak duration. Optimal response strategies substantially mitigated these effects, consistently reducing the number of infected cattle, spatial extent, secondary transmission events, and the overall outbreak timeline compared to suboptimal responses. However, interaction effects between response strategy and preclinical infectiousness varied by region. In the Eastern and Central regions, the beneficial impact of optimal responses became increasingly pronounced as the preclinical infectious period lengthened, significantly attenuating outbreak severity across all metrics. In contrast, in the Western region, while optimal response strategies consistently reduced outbreak severity, their effectiveness showed less dependence on the duration of preclinical infectiousness, except for cumulative spatial spread and peak transmission timing, where longer infectious periods still amplified outbreak impacts under suboptimal conditions.

The marked impact of incubation phase transmission observed in our simulations highlights the vulnerability of current detection and surveillance systems. Specifically, the expected scenario of a suboptimal detection markedly increased epidemic severity, underscoring the necessity for robust early-warning surveillance measures. The early identification of FMD cases is paramount to reducing disease spread, as preclinical transmission events significantly amplify the scope and complexity of outbreaks. The regional variability observed suggests that geographical context, possibly influenced by landscape structure, farm density, or livestock management practices, plays a role in determining outbreak severity and response efficacy. Differences in agricultural demographics and production practices are of particular consideration when generalizing these results to other countries. Tailoring intervention strategies based on regional characteristics, while also preparing for varying durations of preclinical infectiousness, may significantly improve outbreak control and management outcomes.

### 4.2 Low-virulence findings

Within the vast diversity of FMDV, there are known to be strains that have mild clinical signs (low-virulence) or that are completely subclinical in one or more species. The purpose of modeling outbreaks involving a milder strain of FMDV was based on the premise that such strains would be expected to have a longer preclinical phase, which might augment the effects already described for preclinical transmission amongst highly virulent strains. For example, mild signs of FMD in infected sheep was associated with wide virus dissemination during early stages of the 2001 epidemic in the UK (49), and mild or absent lesions have been described during FMDV infection in *Bos indicus* cattle breeds that are common in FMD-endemic regions (50). Furthermore, the strain-specific variability in incubation phase transmission dynamics documented herein echoes findings documented previously (26, 33). The notably prolonged incubation and preclinical infectious phases associated with low-virulence strains, such as strain A/SKR/2010, present unique challenges to outbreak containment efforts (48). When cattle are infected with a biologically less virulent virus strain, detection of infected animals is more difficult due to low-grade clinical signs. These signs may not resemble the classical presentation of FMD or may be slower to present, leading to a delay in initial detection of the disease. The prolonged preclinical infectious phase also complicates contact tracing and surveillance activities. Our simulations predict that transmission of low-virulence strains during the preclinical phase may result in substantial outbreaks affecting millions of cattle and exceeding nine months in duration. These findings highlight the necessity of tailored contingency plans for managing distinct viral phenotypes. Such nuanced insights into strain-specific transmission dynamics could significantly enhance preparedness models and outbreak response protocols.

Our findings substantiate the critical importance of incubation phase transmission in modeling FMDV in cattle, as has been demonstrated more explicitly in pigs. Including a distinct period of preclinical transmission in models of FMDV-spread significantly enhances their predictive accuracy and practical utility. Future research and policy initiatives must prioritize improving early detection, robust surveillance systems, and effective biosecurity measures to mitigate the heightened transmission risks associated with preclinical infectiousness. Addressing these epidemiological realities with targeted preparedness and response strategies would bolster the resilience of livestock production systems against future FMD incursions.

## 5 Conclusion

This work incorporated laboratory data into an epidemiologic model of FMD-spread in U.S. cattle for the purpose of investigating the potential impact of preclinical transmission of FMDV. The impacts of incubation phase transmission were compared under optimal and suboptimal detection scenarios and across various durations of preclinical transmission for three regions of the country. Scenarios which included incubation phase transmission consistently contained more farms with longer outbreak durations compared to scenarios that did not include a distinct period of preclinical transmission. The addition of a single day of incubation phase transmission had a significant impact on outbreak outcomes, even when detection was optimal. However, the impacts of incubation phase transmission were profound in the more realistic scenario when detection was suboptimal, demonstrating that these impacts are influenced by response measures.

The results of this work demonstrate that a distinct preclinical infectious phase should be included in models of FMDV-spread. While prior work in high-containment laboratory settings has demonstrated that incubation phase transmission is a biological reality, applying this data to transmission model shows that preclinical transmission has a significant impact on outbreak outcomes. If epidemiologic models do not include incubation phase transmission, they may underestimate the size and duration of an outbreak, thereby leading to inadequate preparedness for outbreaks in FMD-free regions. These findings support the claim that a distinct period of incubation phase transmission should be included in models of FMDV-spread.

## Data Availability

The datasets presented in this study can be found in online repositories. The names of the repository/repositories and accession number(s) can be found in the article/Supplementary material.
