# Supplementary material for: Epidemiologic consequences of preclinical transmission of foot-and-mouth disease virus in cattle
Source: Front Vet Sci. 2025 Aug 29;12:1651091. doi: 10.3389/fvets.2025.1651091 (PMC12426179; doi:10.3389/fvets.2025.1651091)
Supplement: Supplementary file 1 [file Data_Sheet_1.PDF]

# *Supplementary Material*

## 1 Supplementary Tables

**Supplementary Table 1.** Three regional modeling scenarios were developed to quantify the impacts of incubation phase transmission of FMDV in cattle in multiple regions of the United States. Column headers include the regional scenario name and the number of states in the specified region (*n*). The associated states and the number of cattle premises in the model population file are listed in columns by regional scenario.

| **Regional scenarios and associated states** | | | | | |
|---|---|---|---|---|---|
| **Western** *n = 17* | | **Central** *n = 23* | | **Eastern** *n = 31* | |
| Arizona | 5,796 | Alabama | 21,095 | Alabama | 21,095 |
| California | 16,090 | Arkansas | 25,925 | Arkansas | 25,925 |
| Colorado | 13,974 | Illinois | 16,299 | Connecticut | 1,363 |
| Idaho | 10,705 | Indiana | 16,576 | Delaware | 397 |
| Kansas | 27,431 | Iowa | 27,025 | Florida | 21,263 |
| Montana | 11,906 | Kansas | 27,431 | Georgia | 17,347 |
| Nebraska | 23,292 | Kentucky | 39,793 | Illinois | 16,299 |
| Nevada | 1,825 | Louisiana | 13,666 | Indiana | 16,576 |
| New Mexico | 12,674 | Michigan | 12,741 | Iowa | 27,025 |
| North Dakota | 9,857 | Minnesota | 21,400 | Kentucky | 39,793 |
| Oklahoma | 50,780 | Mississippi | 15,895 | Louisiana | 13,666 |
| Oregon | 14,261 | Missouri | 52,923 | Maine | 2,145 |
| South Dakota | 15,598 | Nebraska | 23,292 | Maryland | 3,298 |
| Texas | 151,215 | New York | 11,046 | Massachusetts | 1,309 |
| Utah | 8,573 | North Dakota | 9,857 | Michigan | 12,741 |
| Washington | 11,548 | Ohio | 23,743 | Minnesota | 21,400 |
| Wyoming | 6,107 | Oklahoma | 50,780 | Mississippi | 15,895 |
| | | Pennsylvania | 21,563 | Missouri | 52,923 |
| | | South Dakota | 15,598 | New Hampshire | 981 |
| | | Tennessee | 38,689 | New Jersey | 1,212 |
| | | Texas | 151,215 | New York | 11,046 |
| | | West Virginia | 12,004 | North Carolina | 19,468 |
| | | Wisconsin | 24,761 | Ohio | 23,743 |
| | | | | Pennsylvania | 21,563 |
| | | | | Rhode Island | 285 |
| | | | | South Carolina | 8,119 |
| | | | | Tennessee | 38,689 |
| | | | | Vermont | 2,254 |
| | | | | Virginia | 23,542 |
| | | | | West Virginia | 12,004 |
| | | | | Wisconsin | 24,761 |
| **Western total** | 391,632 | **Central total** | 673,317 | **Eastern total** | 498,127 |

**Supplementary Table 2.** Infectivity parameters used to simulate preclinical transmission of FMDV between cattle herds in InterSpread Plus. Infectivity parameters describe the infectivity of a farm, on a scale from 0 to 1, over time, from the onset of infection. The inputs for the low-virulence scenarios and those with no preclinical transmission (baseline) were identical because these scenarios shared a 4-day latent period and therefore, became infectious on the same day relative to infection.

| **Infectivity of cattle farms on a scale of 0 to 1, over time, from the onset of infection** | | | | |
|---|---|---|---|---|
| **Row 1:** Days following onset of infection | | | | |
| **Row 2:** Infectivity on a scale of 0 to 1 | | | | |
| **No preclinical transmission** | | | | |
| 0 | 3 | 4 | 60 | 1000 |
| 0 | 0 | 1 | 1 | 0 |
| **1-day preclinical infectious duration** | | | | |
| 0 | 2 | 3 | 60 | 1000 |
| 0 | 0 | 1 | 1 | 0 |
| **2-day preclinical infectious duration** | | | | |
| 0 | 1 | 2 | 60 | 1000 |
| 0 | 0 | 1 | 1 | 0 |
| **3-day preclinical infectious duration** | | | | |
| 0 | 1 | 60 | 1000 | |
| 0 | 1 | 1 | 0 | |
| **Low-virulence** | | | | |
| 0 | 3 | 4 | 60 | 1000 |
| 0 | 0 | 1 | 1 | 0 |

**Supplementary Table 3.** Parameters used to simulate detection of FMDV in cattle herds via active surveillance in zones around detected premises in InterSpread Plus. The table specifies the probability of a farm being detected relative to the onset of infection. Detection probabilities varied by scenario to simulate detection of farms prior to the onset of clinical signs in scenarios that included preclinical transmission of FMDV.

| **Probability of detection via contact tracing and active surveillance in zones** | | | | | |
|---|---|---|---|---|---|
| **Row 1:** Days following onset of infection | | | | | |
| **Row 2:** Probability of detection | | | | | |
| **No preclinical transmission** | | | | | |
| 0 | 1 | 2 | 3 | 4 | 43 |
| 0 | 0 | 0 | 0 | 0.97 | 0.97 |
| **1-day preclinical infectious duration** | | | | | |
| 0 | 1 | 2 | 3 | 4 | 43 |
| 0 | 0 | 0 | 0.7275 | 0.97 | 0.97 |
| **2-day preclinical infectious duration** | | | | | |
| 0 | 1 | 2 | 3 | 4 | 43 |
| 0 | 0 | 0.7275 | 0.7275 | 0.97 | 0.97 |
| **3-day preclinical infectious duration** | | | | | |
| 0 | 1 | 2 | 3 | 4 | 43 |
| 0 | 0.7275 | 0.7275 | 0.7275 | 0.97 | 0.97 |
| **Low-virulence** | | | | | |
| 0 | 3 | 4 | 9 | 10 | 43 |
| 0 | 0 | 0.7275 | 0.7275 | 0.97 | 0.97 |

**Supplementary Table 4.** Parameters used to simulate surveillance activities in InterSpread Plus. The 'delay to detect' is a probability distribution returning the number of days from when the surveillance activity took place to when the premises is detected. The 'delay to detect' varied between optimal and suboptimal detection scenarios. The low-virulence scenarios used the same probability distributions as the suboptimal detection scenarios.

| **Delay to detect via passive surveillance, active surveillance, and contact tracing** | | |
|---|---|---|
| **Surveillance activity** | **Optimal detection** | **Suboptimal detection and low-virulence** |
| Passive surveillance | BetaPert 1 1 2 | BetaPert 2 2 3 |
| Active surveillance in zones | BetaPert 0 1 2 | BetaPert 1 2 3 |
| Contact tracing | BetaPert 0 1 2 | BetaPert 1 2 3 |
