# Supplementary material for: Epidemiologic consequences of preclinical transmission of foot-and-mouth disease virus in cattle
Source: Front Vet Sci. 2025 Aug 29;12:1651091. doi: 10.3389/fvets.2025.1651091 (PMC12426179; doi:10.3389/fvets.2025.1651091)
Supplement: Supplementary file 2 [file Data_Sheet_2.PDF]

Supplementary Material

2 Probability of Transmission Tables for InterSpread Plus

Supplementary Table 5. Parameters used to simulate transmission of FMDV between cattle farms in InterSpread Plus via direct contacts. Inputs specify the probability that a farm will transmit FMDV, over time, from the onset of infection.

**No preclinical transmission**									
Probability of transmission from direct contacts originating from cow calf premises on which disease has not been detected									
**Row 1:** Days following onset of infection									
**Row 2:** Probability of transmission									
**Origin:** cow calf [large capacity] [Western U.S.]									
0	3	4	19	20	21	22	23	24	
0	0	1	1	0.9734	0.3789	0.2117	0.2117	0	
**Origin:** cow calf [large capacity] [Eastern U.S.]									
0	3	4	18	19	20	21	23	24	
0	0	1	1	0.9990	0.7747	0.1369	0.1369	0	
**Origin:** cow calf [small capacity] [Western U.S.]									
0	3	4	13	14	15	16	17	18	20
0	0	1	1	0.9989	0.9858	0.8781	0.538	0.1351	0.1351
21									
0									
**Origin:** cow calf [small capacity] [Eastern U.S.]									
0	3	4	13	14	15	16	17	18	20
0	0	1	1	0.9999	0.9951	0.9171	0.5464	0.1714	0.1714
21									
0									

Probability of transmission from direct contacts originating from dairy premises on which disease has not been detected									
**Row 1:** Days following onset of infection									
**Row 2:** Probability of transmission									
**Origin:** dairy [large capacity] [all regions]									
0	3	4	13	14	15	16	17	18	19
0	0	1	1	0.9999	0.9999	0.9843	0.6380	0.0378	0.0055
20									
0									

No preclinical transmission, continued

Probability of transmission from direct contacts originating from dairy premises on which disease has not been detected

Row 1: Days following onset of infection

Row 2: Probability of transmission

Origin: dairy [small capacity] [all regions]

0	3	4	5	6	12	13	14	15	16
0	0	0.9988	0.9999	1	1	0.9999	0.9994	0.9891	0.8990
17	18	19	21	22					
0.5812	0.2198	0.0588	0.0588	0					

Origin: dairy heifer calf [large capacity] [all regions]

0	3	4	5	6	7	8	9	10	11
0	0	0.8707	0.9891	0.9999	1	0.9999	0.9999	0.9998	0.9985
12	13	14	15	16	17	18	19	20	
0.9916	0.9632	0.8763	0.6860	0.4029	0.1418	0.0190	0.0013	0	

Origin: dairy heifer calf [medium capacity] [all regions]

0	3	4	5	6	7	9	10	11	12
0	0	0.9957	0.9999	1	0.9999	0.9999	0.9992	0.9955	0.9812
13	14	15	16	17	18	19			
0.9367	0.8232	0.6052	0.3164	0.0835	0.0005	0			

Origin: dairy heifer calf [small capacity] [all regions]

0	3	4	5	6	7	8	9	10	11
0	0	0.8153	0.9427	0.9862	0.9996	0.9999	0.9997	0.9977	0.9908
12	13	14	15	16	17	18	19	20	21
0.9719	0.9270	0.8344	0.6662	0.4195	0.1755	0.0442	0.0116	0.0019	0

Probability of transmission from direct contacts originating from cattle feedlots and stockers on which disease has not been detected

Row 1: Days following onset of infection

Row 2: Probability of transmission

Origin: feedlot [large & medium capacity] [all regions]

0	3	4	5	6	19	20	21	22	23
0	0	0.5651	0.9665	1	1	0.8969	0.0670	0.0013	0

Origin: feedlot [small capacity] [all regions]

0	3	4	5	13	14	15	16	17	18
0	0	0.9999	1	1	0.9992	0.9843	0.8586	0.4657	0.1175
19	20	21	22						
0.0347	0.0175	0.0175	0						

No preclinical transmission, continued

Probability of transmission from direct contacts originating from cattle feedlots and stockers on which disease has not been detected

Row 1: Days following onset of infection

Row 2: Probability of transmission

Origin: feedlot [large & medium capacity] [all regions]

0	3	4	5	6	19	20	21	22	23
0	0	0.5651	0.9665	1	1	0.8969	0.0670	0.0013	0

Origin: feedlot [small capacity] [all regions]

0	3	4	5	13	14	15	16	17	18
0	0	0.9999	1	1	0.9992	0.9843	0.8586	0.4657	0.1175
19	20	21	22						
0.0347	0.0175	0.0175	0						

Origin: stocker [large capacity] [Western, Central and Northeast U.S.]

0	3	4	19	20	21	22	23		
0	0	1	1	0.9642	0.1078	0.0554	0		

Origin: stocker [large capacity] [Southeast U.S.]

0	3	4	19	20	21	22			
0	0	1	1	0.6671	0.0552	0			

Origin: stocker [small capacity] [Western, Central and Northeast U.S.]

0	3	4	15	16	17	18	19	21	22
0	0	1	1	0.9976	0.8570	0.3348	0.1831	0.1831	0

Origin: stocker [small capacity] [Southeast U.S.]

0	3	4	14	15	16	17	18	20	21
0	0	1	1	0.9998	0.9791	0.7170	0.1800	0.1800	0

Probability of transmission from direct contacts originating from cattle dealers and livestock markets on which disease has not been detected

Row 1: Days following onset of infection

Row 2: Probability of transmission

Origin: dealer [all regions]

0	3	4	16	17	18	20	21		
0	0	1	1	0.9946	0.4500	0.4500	0		

Origin: livestock market [all regions]

0	3	4	5	11	12	13	14	15	16
0	0	0.9996	1	1	0.9999	0.9997	0.9987	0.9903	0.9227
17	18	19	20	21	22	23	24		
0.6672	0.2498	0.1867	0.1819	0.0474	0.0089	0.0087	0		

**1-day preclinical infectious duration**									
Probability of transmission from direct contacts originating from cow calf premises on which disease has not been detected									
**Row 1:** Days following onset of infection									
**Row 2:** Probability of transmission									
**Origin:** cow calf [large capacity] [Western U.S.]									
0	2	3	4	19	20	21	22	23	24
0	0	0.9873	1	1	0.9734	0.3789	0.2117	0.2117	0
**Origin:** cow calf [large capacity] [Eastern U.S.]									
0	2	3	4	18	19	20	21	23	24
0	0	0.9225	1	1	0.9990	0.7747	0.1369	0.1369	0
**Origin:** cow calf [small capacity] [Western U.S.]									
0	2	3	4	13	14	15	16	17	18
0	0	0.9228	1	1	0.9989	0.9858	0.8781	0.538	0.1351
20	21								
0.1351	0								
**Origin:** cow calf [small capacity] [Eastern U.S.]									
0	2	3	4	13	14	15	16	17	18
0	0	0.9540	1	1	0.9999	0.9951	0.9171	0.5464	0.1714
20	21								
0.1714	0								

Probability of transmission from direct contacts originating from dairy premises on which disease has not been detected									
**Row 1:** Days following onset of infection									
**Row 2:** Probability of transmission									
**Origin:** dairy [large capacity] [all regions]									
0	2	3	4	13	14	15	16	17	18
0	0	0.9614	1	1	0.9999	0.9999	0.9843	0.6380	0.0378
19	20								
0.0055	0								
**Origin:** dairy [small capacity] [all regions]									
0	2	3	4	5	6	12	13	14	15
0	0	0.6117	0.9988	0.9999	1	1	0.9999	0.9994	0.9891
16	17	18	19	21	22				
0.8990	0.5812	0.2198	0.0588	0.0588	0				
**Origin:** dairy heifer calf [large capacity] [all regions]									
0	2	3	4	5	6	7	8	9	10
0	0	0.1736	0.8707	0.9891	0.9999	1	0.9999	0.9999	0.9998
11	12	13	14	15	16	17	18	19	20
0.9985	0.9916	0.9632	0.8763	0.6860	0.4029	0.1418	0.0190	0.0013	0

1-day preclinical infectious duration, continued

Probability of transmission from direct contacts originating from dairy premises on which disease has not been detected

Row 1: Days following onset of infection

Row 2: Probability of transmission

Origin: dairy heifer calf [medium capacity] [all regions]

0	2	3	4	5	6	7	9	10	11
0	0	0.3230	0.9957	0.9999	1	0.9999	0.9999	0.9992	0.9955
12	13	14	15	16	17	18	19		
0.9812	0.9367	0.8232	0.6052	0.3164	0.0835	0.0005	0		

Origin: dairy heifer calf [small capacity] [all regions]

0	2	3	4	5	6	7	8	9	10
0	0	0.1767	0.8153	0.9427	0.9862	0.9996	0.9999	0.9997	0.9977
11	12	13	14	15	16	17	18	19	20
0.9908	0.9719	0.9270	0.8344	0.6662	0.4195	0.1755	0.0442	0.0116	0.0019
21									
0									

Probability of transmission from direct contacts originating from cattle feedlots and stockers on which disease has not been detected

Row 1: Days following onset of infection

Row 2: Probability of transmission

Origin: feedlot [large & medium capacity] [all regions]

0	2	3	4	5	6	19	20	21	22
0	0	0.0592	0.5651	0.9665	1	1	0.8969	0.0670	0.0013
23									
0									

Origin: feedlot [small capacity] [all regions]

0	2	3	4	5	13	14	15	16	17
0	0	0.6539	0.9999	1	1	0.9992	0.9843	0.8586	0.4657
18	19	20	21	22					
0.1175	0.0347	0.0175	0.0175	0					

Origin: stocker [large capacity] [Western, Central, and Northeast U.S.]

0	2	3	4	19	20	21	22	23
0	0	0.9916	1	1	0.9642	0.1078	0.0554	0

Origin: stocker [large capacity] [Southeast U.S.]

0	2	3	4	19	20	21	22
0	0	0.9777	1	1	0.6671	0.0552	0

1-day preclinical infectious duration, continued									
Probability of transmission from direct contacts originating from cattle feedlots and stockers on which disease has not been detected									
**Row 1:** Days following onset of infection									
**Row 2:** Probability of transmission									
**Origin:** stocker [small capacity] [Western, Central, and Northeast U.S.]									
0	2	3	4	15	16	17	18	19	21
0	0	0.9967	1	1	0.9976	0.8570	0.3348	0.1831	0.1831
22									
0									
**Origin:** stocker [small capacity] [Southeast U.S.]									
0	2	3	4	14	15	16	17	18	20
0	0	0.9791	1	1	0.9998	0.9791	0.7170	0.1800	0.1800
21									
0									

Probability of transmission from direct contacts originating from cattle dealers and livestock markets on which disease has not been detected									
**Row 1:** Days following onset of infection									
**Row 2:** Probability of transmission									
**Origin:** dealer [all regions]									
0	2	3	4	16	17	18	20	21	
0	0	1	1	1	0.9946	0.4500	0.4500	0	
**Origin:** livestock market [all regions]									
0	2	3	4	5	11	12	13	14	15
0	0	0.8731	0.9996	1	1	0.9999	0.9997	0.9987	0.9903
16	17	18	19	20	21	22	23	24	
0.9227	0.6672	0.2498	0.1867	0.1819	0.0474	0.0089	0.0087	0	

2-day preclinical infectious duration									
Probability of transmission from direct contacts originating from cow calf premises on which disease has not been detected									
**Row 1:** Days following onset of infection									
**Row 2:** Probability of transmission									
**Origin:** cow calf [large capacity] [Western U.S.]									
0	1	2	3	4	19	20	21	22	23
0	0	0.6972	0.9873	1	1	0.9734	0.3789	0.2117	0.2117
24									
0									

**2-day preclinical infectious duration, continued**									
Probability of transmission from direct contacts originating from cow calf premises on which disease has not been detected									
**Row 1:** Days following onset of infection									
**Row 2:** Probability of transmission									
**Origin:** cow calf [large capacity] [Eastern U.S.]									
0	1	2	3	4	18	19	20	21	23
0	0	0.5230	0.9225	1	1	0.9990	0.7747	0.1369	0.1369
24									
0									
**Origin:** cow calf [small capacity] [Western U.S.]									
0	1	2	3	4	13	14	15	16	17
0	0	0.7745	0.9228	1	1	0.9989	0.9858	0.8781	0.538
18	20	21							
0.1351	0.1351	0							
**Origin:** cow calf [small capacity] [Eastern U.S.]									
0	1	2	3	4	13	14	15	16	17
0	0	0.8176	0.9540	1	1	0.9999	0.9951	0.9171	0.5464
18	20	21							
0.1714	0.1714	0							

Probability of transmission from direct contacts originating from dairy premises on which disease has not been detected									
**Row 1:** Days following onset of infection									
**Row 2:** Probability of transmission									
**Origin:** dairy [large capacity] [all regions]									
0	1	2	3	4	13	14	15	16	17
0	0	0.1144	0.9614	1	1	0.9999	0.9999	0.9843	0.6380
18	19	20							
0.0378	0.0055	0							
**Origin:** dairy [small capacity] [all regions]									
0	1	2	3	4	5	6	12	13	14
0	0	0.4012	0.6117	0.9988	0.9999	1	1	0.9999	0.9994
15	16	17	18	19	21	22			
0.9891	0.8990	0.5812	0.2198	0.0588	0.0588	0			

**2-day preclinical infectious duration, continued**									
Probability of transmission from direct contacts originating from dairy premises on which disease has not been detected									
**Row 1:** Days following onset of infection									
**Row 2:** Probability of transmission									
**Origin:** dairy heifer calf [large capacity] [all regions]									
0	1	2	3	4	5	6	7	8	9
0	0	0.0042	0.1736	0.8707	0.9891	0.9999	1	0.9999	0.9999
10	11	12	13	14	15	16	17	18	19
0.9998	0.9985	0.9916	0.9632	0.8763	0.6860	0.4029	0.1418	0.0190	0.0013
20									
0									
**Origin:** dairy heifer calf [medium capacity] [all regions]									
0	1	2	3	4	5	6	7	9	10
0	0	0.0358	0.3230	0.9957	0.9999	1	0.9999	0.9999	0.9992
11	12	13	14	15	16	17	18	19	
0.9955	0.9812	0.9367	0.8232	0.6052	0.3164	0.0835	0.0005	0	
**Origin:** dairy heifer calf [small capacity] [all regions]									
0	1	2	3	4	5	6	7	8	9
0	0	0.0822	0.1767	0.8153	0.9427	0.9862	0.9996	0.9999	0.9997
10	11	12	13	14	15	16	17	18	19
0.9977	0.9908	0.9719	0.9270	0.8344	0.6662	0.4195	0.1755	0.0442	0.0116
20	21								
0.0019	0								

Probability of transmission from direct contacts originating from cattle feedlots and stockers on which disease has not been detected									
**Row 1:** Days following onset of infection									
**Row 2:** Probability of transmission									
**Origin:** feedlot [large & medium capacity] [all regions]									
0	1	2	3	4	5	6	19	20	21
0	0	0.0032	0.0592	0.5651	0.9665	1	1	0.8969	0.0670
22	23								
0.0013	0								
**Origin:** feedlot [small capacity] [all regions]									
0	1	2	3	4	5	13	14	15	16
0	0	0.3079	0.6539	0.9999	1	1	0.9992	0.9843	0.8586
17	18	19	20	21	22				
0.4657	0.1175	0.0347	0.0175	0.0175	0				

**2-day preclinical infectious duration, continued**									
Probability of transmission from direct contacts originating from cattle feedlots and stockers on which disease has not been detected									
**Row 1:** Days following onset of infection									
**Row 2:** Probability of transmission									
**Origin:** stocker [large capacity] [Western, Central, and Northeast U.S.]									
0	1	2	3	4	19	20	21	22	23
0	0	0.3293	0.9916	1	1	0.9642	0.1078	0.0554	0
**Origin:** stocker [large capacity] [Southeast U.S.]									
0	1	2	3	4	19	20	21	22	
0	0	0.2475	0.9777	1	1	0.6671	0.0552	0	
**Origin:** stocker [small capacity] [Western, Central, and Northeast U.S.]									
0	1	2	3	4	15	16	17	18	19
0	0	0.9579	0.9967	1	1	0.9976	0.8570	0.3348	0.1831
21	22								
0.1831	0								
**Origin:** stocker [small capacity] [Southeast U.S.]									
0	1	2	3	4	14	15	16	17	18
0	0	0.8909	0.9791	1	1	0.9998	0.9791	0.7170	0.1800
20	21								
0.1800	0								

Probability of transmission from direct contacts originating from cattle dealers and livestock markets on which disease has not been detected									
**Row 1:** Days following onset of infection									
**Row 2:** Probability of transmission									
**Origin:** dealer [all regions]									
0	1	2	3	4	16	17	18	20	21
0	0	0.9889	1	1	1	0.9946	0.4500	0.4500	0
**Origin:** livestock market [all regions]									
0	1	2	3	4	5	11	12	13	14
0	0	0.6853	0.8731	0.9996	1	1	0.9999	0.9997	0.9987
15	16	17	18	19	20	21	22	23	24
0.9903	0.9227	0.6672	0.2498	0.1867	0.1819	0.0474	0.0089	0.0087	0

3-day preclinical infectious duration

Probability of transmission from direct contacts originating from cow calf premises on which disease has not been detected

Row 1: Days following onset of infection

Row 2: Probability of transmission

Origin: cow calf [large capacity] [Western U.S.]

0	1	2	3	4	19	20	21	22	23
0	0.6972	0.6972	0.9873	1	1	0.9734	0.3789	0.2117	0.2117
24									
0									

Origin: cow calf [large capacity] [Eastern U.S.]

0	1	2	3	4	18	19	20	21	23
0	0.5230	0.5230	0.9225	1	1	0.9990	0.7747	0.1369	0.1369
24									
0									

Origin: cow calf [small capacity] [Western U.S.]

0	1	2	3	4	13	14	15	16	17
0	0.7745	0.7745	0.9228	1	1	0.9989	0.9858	0.8781	0.538
18	20	21							
0.1351	0.1351	0							

Origin: cow calf [small capacity] [Eastern U.S.]

0	1	2	3	4	13	14	15	16	17
0	0.8176	0.8176	0.9540	1	1	0.9999	0.9951	0.9171	0.5464
18	20	21							
0.1714	0.1714	0							

Probability of transmission from direct contacts originating from dairy premises on which disease has not been detected

Row 1: Days following onset of infection

Row 2: Probability of transmission

Origin: dairy [large capacity] [all regions]

0	1	2	3	4	13	14	15	16	17
0	0.0378	0.1144	0.9614	1	1	0.9999	0.9999	0.9843	0.6380
18	19	20							
0.0378	0.0055	0							

Origin: dairy [small capacity] [all regions]

0	1	2	3	4	5	6	12	13	14
0	0.4012	0.4012	0.6117	0.9988	0.9999	1	1	0.9999	0.9994
15	16	17	18	19	21	22			
0.9891	0.8990	0.5812	0.2198	0.0588	0.0588	0			

3-day preclinical infectious duration, continued

Probability of transmission from direct contacts originating from dairy premises on which disease has not been detected

Row 1: Days following onset of infection

Row 2: Probability of transmission

Origin: dairy heifer calf [large capacity] [all regions]

0	1	2	3	4	5	6	7	8	9
0	0.0011	0.0042	0.1736	0.8707	0.9891	0.9999	1	0.9999	0.9999
10	11	12	13	14	15	16	17	18	19
0.9998	0.9985	0.9916	0.9632	0.8763	0.6860	0.4029	0.1418	0.0190	0.0013
20									
0									

Origin: dairy heifer calf [medium capacity] [all regions]

0	1	2	3	4	5	6	7	9	10
0	0.0238	0.0358	0.3230	0.9957	0.9999	1	0.9999	0.9999	0.9992
11	12	13	14	15	16	17	18	19	
0.9955	0.9812	0.9367	0.8232	0.6052	0.3164	0.0835	0.0005	0	

Origin: dairy heifer calf [small capacity] [all regions]

0	1	2	3	4	5	6	7	8	9
0	0.0809	0.0822	0.1767	0.8153	0.9427	0.9862	0.9996	0.9999	0.9997
10	11	12	13	14	15	16	17	18	19
0.9977	0.9908	0.9719	0.9270	0.8344	0.6662	0.4195	0.1755	0.0442	0.0116
20	21								
0.0019	0								

Probability of transmission from direct contacts originating from cattle feedlots and stockers on which disease has not been detected

Row 1: Days following onset of infection

Row 2: Probability of transmission

Origin: feedlot [large & medium capacity] [all regions]

0	1	2	3	4	5	6	19	20	21
0	0.0026	0.0032	0.0592	0.5651	0.9665	1	1	0.8969	0.0670
22	23								
0.0013	0								

Origin: feedlot [small capacity] [all regions]

0	1	2	3	4	5	13	14	15	16
0	0.2945	0.3079	0.6539	0.9999	1	1	0.9992	0.9843	0.8586
17	18	19	20	21	22				
0.4657	0.1175	0.0347	0.0175	0.0175	0				

3-day preclinical infectious duration, continued

Probability of transmission from direct contacts originating from cattle feedlots and stockers on which disease has not been detected

Row 1: Days following onset of infection

Row 2: Probability of transmission

Origin: stocker [large capacity] [Western, Central, and Northeast U.S.]

0	1	2	3	4	19	20	21	22	23
0	0.2899	0.3293	0.9916	1	1	0.9642	0.1078	0.0554	0

Origin: stocker [large capacity] [Southeast U.S.]

0	1	2	3	4	19	20	21	22
0	0.2475	0.2475	0.9777	1	1	0.6671	0.0552	0

Origin: stocker [small capacity] [Western, Central, and Northeast U.S.]

0	1	2	3	4	15	16	17	18	19
0	0.9579	0.9579	0.9967	1	1	0.9976	0.8570	0.3348	0.1831
21	22								
0.1831	0								

Origin: stocker [small capacity] [Southeast U.S.]

0	1	2	3	4	14	15	16	17	18
0	0.8909	0.8909	0.9791	1	1	0.9998	0.9791	0.7170	0.1800
20	21								
0.1800	0								

Probability of transmission from direct contacts originating from cattle dealers and livestock markets on which disease has not been detected

Row 1: Days following onset of infection

Row 2: Probability of transmission

Origin: dealer [all regions]

0	1	2	3	4	16	17	18	20	21
0	0.9889	0.9889	1	1	1	0.9946	0.4500	0.4500	0

Origin: livestock market [all regions]

0	1	2	3	4	5	11	12	13	14
0	0.6804	0.6853	0.8731	0.9996	1	1	0.9999	0.9997	0.9987
15	16	17	18	19	20	21	22	23	24
0.9903	0.9227	0.6672	0.2498	0.1867	0.1819	0.0474	0.0089	0.0087	0

Supplementary Table 6. Parameters used to simulate preclinical transmission of FMDV between cattle farms in InterSpread Plus via indirect contacts. Inputs specify the probability that a farm will transmit FMDV, over time, from the onset of infection. Parameters for preclinical transmission vary by scenario.

**Transmission from preclinical premises**				
Probability of transmission from indirect, **medium-risk** contacts originating from cattle premises that are undetected and preclinical				
**Row 1:** Days following onset of **infection**				
**Row 2:** Probability of transmission				
**No preclinical transmission**				
0	1	2	3	4
0	0	0	0	0
**1-day preclinical infectious duration**				
0	1	2	3	4
0	0	0	0.1	0
**2-day preclinical infectious duration**				
0	1	2	3	4
0	0	0.1	0.1	0
**3-day preclinical infectious duration**				
0	1	2	3	4
0	0.1	0.1	0.1	0

Probability of transmission from indirect, **low-risk** contacts originating from cattle premises that are undetected and preclinical				
**Row 1:** Days following onset of **infection**				
**Row 2:** Probability of transmission				
**No preclinical transmission**				
0	1	2	3	4
0	0	0	0	0
**1-day preclinical infectious duration**				
0	1	2	3	4
0	0	0	0.02	0
**2-day preclinical infectious duration**				
0	1	2	3	4
0	0	0.02	0.02	0
**3-day preclinical infectious duration**				
0	1	2	3	4
0	0.02	0.02	0.02	0

Transmission from preclinical premises, continued				
Probability of transmission from indirect contacts originating from cattle premises that are **detected** and preclinical				
**Row 1:** Days following onset of **infection**				
**Row 2:** Probability of transmission				
**No preclinical transmission**				
0	1	2	3	4
0	0	0	0	0
**1-day preclinical infectious duration**				
0	1	2	3	4
0	0	0	0.01	0
**2-day preclinical infectious duration**				
0	1	2	3	4
0	0	0.01	0.01	0
**3-day preclinical infectious duration**				
0	1	2	3	4
0	0.01	0.01	0.01	0

Supplementary Table 7. Parameters used to simulate transmission of FMDV between cattle farms in InterSpread Plus via indirect contacts during the clinical phase. Inputs specify the probability that a farm will transmit FMDV, over time, from clinical onset. These parameters were consistent for all scenarios.

**Transmission from clinical premises**							
Probability of transmission from indirect, **medium-risk** contacts originating from cattle premises that are undetected and expressing clinical signs							
**Row 1:** Days following onset of **clinical signs**							
**Row 2:** Probability of transmission							
0	1	2	6	7	11	12	16
0.1	0.1	0.2	0.2	0.4	0.4	0.5	0.5
17	21	22	26	27			
0.4	0.4	0.2	0.2	0.1			

Probability of transmission from indirect, **low-risk** contacts originating from cattle premises that are undetected and expressing clinical signs							
**Row 1:** Days following onset of **clinical signs**							
**Row 2:** Probability of transmission							
0	1	2	6	7	11	12	16
0.02	0.02	0.04	0.04	0.08	0.08	0.1	0.1
17	21	22	26	27			
0.08	0.08	0.04	0.04	0.02			
